# Targeting deeply-sourced seeps along the Central Volcanic Zone

**DOI:** 10.12688/openreseurope.17806.1

**Published:** 2024-10-18

**Authors:** Deborah Bastoni, Mauricio Aguilera, Felipe Aguilera, Jenny M. Blamey, Joy Buongiorno, Agostina Chiodi, Angelina Cordone, Alfredo Esquivel, Marco Giardina, Cristobal Gonzalez, Joaquin Gutierrez, Nahun Irarrazabal, Viola Krukenberg, Susana Layana, Jacopo Pasotti, Carlos J. Ramirez, Alejandro Rodriguez, Timothy J. Rogers, Claudia Rojas, Jorge Sánchez-SanMartín, Matt O. Schrenk, Hector Vallejos, Gerdhard L. Jessen, Peter H. Barry, J. Maarten de Moor, Karen G. Lloyd, Donato Giovannelli

**Affiliations:** 1 Department of Biology, University of Naples Federico II, Naples, Campania, Italy; 2 Millennium Institute on Volcanic Risk Research, Ckelar Volcanoes, Antofagasta, Chile; 3 Programa de Magíster en Ciencias Mención Geología, Universidad Catolica del Norte, Antofagasta, Antofagasta Region, Chile; 4 Departamento de Ciencias Geológicas, Universidad Catolica del Norte, Antofagasta, Antofagasta Region, Chile; 5 Facultad de Química y Biología, Universidad de Santiago de Chile, Santiago, Santiago Metropolitan Region, Chile; 6 Division of Natural Sciences, Maryville College, Maryville, Tennessee, USA; 7 (IBIGEO, UNSa-CONICET), Instituto de Bio y Geociencias del NOA, Salta, Argentina; 8 Department of Chemistry and Biochemistry, Center for Biofilm Engineering, and Thermal Biology Institute, Montana State University, Bozeman, USA; 9 (SeGeoAm), Servicio Geológico Ambiental, Heredia, Costa Rica; 10 Observatorio Vulcanológico y Sismológico de Costa Rica, Universidad Nacional, Heredia, Costa Rica; 11 Department of Microbiology, University of Tennessee, TN, USA; 12 Fundación Biociencia, José Domingo Cañas 2280, Santiago, Chile; 13 Department of Earth and Environmental Sciences, Michigan State University, East Lansing, Michigan, USA; 14 Instituto de Ciencias Marinas y Limnológicas, Universidad Austral de Chile, Valdivia, Los Ríos Region, Chile; 15 Center for Oceanographic Research COPAS COASTAL, Universidad de Concepción, Concepción, Chile; 16 Marine Chemistry & Geochemistry Department, Woods Hole Oceanographic Institution, Woods Hole, Massachusetts, USA; 17 Department of Earth and Planetary Sciences, The University of New Mexico, Albuquerque, New Mexico, USA; 18 Earth Science Department, University of Southern California, Los Angeles, California, USA; 19 Institute for Marine Biological Resources and Biotechnologies, (CNR-IRBIM), National Research Council, Ancona, Italy; 20 Earth-Life Science Institute, Tokyo Institute of Technology, Tokyo, Japan; 21 Department of Marine and Coastal Science, Rutgers University, New Brunswick, New Jersey, USA

**Keywords:** Expedition report, Hot Springs, Andean Convergent Margin, Subduction, Chile, Biogeochemistry

## Abstract

At convergent margins, plates collide producing a subduction process. When an oceanic plate collides with a continental plate, the denser (i.e., oceanic) plate subducts beneath the less dense (continental) plate. This process results in the transportation of carbon and other volatiles into Earth’s deep interior and is counterbalanced by volcanic outgassing. Sampling deeply-sourced seeps and fumaroles throughout a convergent margin allows us to assess the processes that control the inventory of volatiles and their interaction with the deep subsurface microbial communities. The Andean Convergent Margin is volcanically active in four distinct zones: the Northern Volcanic Zone, the Central Volcanic Zone, the Southern Volcanic Zone and the Austral Volcanic Zone, which are each characterised by significantly different subduction parameters like crustal thickness, age of subduction and subduction angle. These differences can change subduction dynamics along the convergent margin, possibly influencing the recycling efficiency of carbon and volatiles and its interaction with the subsurface microbial communities. We carried out a scientific expedition, sampling along a ~800 km convergent margin segment of the Andean Convergent Margin in the Central Volcanic Zone of northern Chile, between 17 °S and 24 °S, sampling fluids, gases and sediments, in an effort to understand interactions between microbiology, deeply-sourced fluids, the crust, and tectonic parameters. We collected samples from 38 different sites, representing a wide diversity of seep types in different geologic contexts. Here we report the field protocols and the descriptions of the sites and samples collected.

## Plain language summary

At convergent margins, where tectonic plates collide, oceanic plates subduct beneath continental plates, carrying carbon and other volatiles into Earth's deep interior. This process is balanced by volcanic outgassing. By sampling deeply-sourced seeps and fumaroles along a convergent margin, scientists can study how volatiles interact with deep subsurface microbial communities. The Andean Convergent Margin, with its distinct volcanic zones, provides an ideal natural laboratory for studying these processes. A scientific expedition along a segment of the Andean Convergent Margin in northern Chile's Central Volcanic Zone aimed to understand these interactions. Samples were collected from 38 sites, representing various seep types in different geologic contexts. This report outlines the field protocols and describes the sites and samples collected.

## Introduction

Along convergent margins, the subduction of one plate underneath another one transports carbon and other volatiles into Earth’s deep interior and is counterbalanced by outgassing ( Kelemen & Manning, 2015) through a series of primary and secondary geothermal emissions. These manifestations release volatile species and elements that contribute to sustaining life ( Barry *et al.*, 2019; Bekaert *et al.*, 2021; Fullerton *et al.*, 2021; Giovannelli *et al.*, 2022a; Rustioni *et al.*, 2021). Variations in subduction parameters, together with the intricate interplay between the descending slab, the overlying mantle wedge, and the overriding lithosphere, collectively exert a profound influence on the thermal and volatile fluxes produced, the nature of the underlying rock formations, and their interactions with percolating fluids, among other factors ( Eberle *et al.*, 2002; Hu & Gurnis, 2020). Previous studies have shown that these changes can influence the subsurface microbial community diversity and its effects on the convergent margin volatile budget ( Barry *et al.*, 2022; Basili *et al.*, 2024; Fullerton *et al.*, 2021; Rogers *et al.*, 2023; Upin *et al.*, 2023). Additionally, changes in trace elements delivered to the surface might be linked to changes in the functional diversity of the microbial community ( Giovannelli, 2023; Hay Mele *et al.*, 2023).

The Andean Convergent Margin (ACM) represents an ocean-continent collision zone characterised by a high degree of seismic and volcanic activity, and elevated heat flow. Here, among the many volcanic manifestations, deeply sourced seeps are invaluable windows into the geological processes linked to past and present tectonic context. These seeps are the superficial manifestation of subsurface fluids, carrying with them geochemical signatures from both the mantle and the crust. While it is established that tectonic processes significantly dictate the location, temperature, and geochemical composition of geothermal fluids and related emissions, our understanding of their impact on microbial taxonomic and functional diversity remains limited. Within this framework, we sampled sediment, fluid and gas samples from 38 deeply sourced seeps and fumaroles along a ~800 km segment within the CVZ of northern Chile (17 °S and 24 °S). This project aims to understand the geochemical transformations and microbial communities in response to various subduction parameters, including crustal thickness and other factors such as carbon input from the slab, upper plate thickness, lithology, and slab dip angles, which can then be used to understand a wide range of global convergent margins. In this report, we present the field protocols alongside detailed descriptions of the sites and samples collected.

## Expedition team and logistics

The expedition team was composed of 27 interdisciplinary scientists spanning the fields of microbiology, biogeochemistry, microbial ecology and gas geochemistry. The sampling was carried out along a segment of the ACM in the CVZ of northern Chile, between 17 °S and 24 °S, during the expedition CH22 conducted between March and April 2022. We collected gas, fluids and sediments from fumaroles, wells, bubbling springs and naturally flowing fluid springs, broadly defined as deeply-sourced seeps ( Giovannelli *et al.*, 2022b), from 38 sites.

## Geological and environmental settings

Situated along the Pacific's "Ring of Fire," Chile is a geologically dynamic region characterised by complex tectonic interactions. Chile stretches over 4,000 kilometres from north to south, encompassing a number of geological terrains that have been shaped through time by plate interactions between the Nazca Plate, subducting under the South American Plate at the Peru–Chile Trench, and the Antarctic Plate. The 7,000-km-long ACM serves as a paradigmatic example of a "typical" of long-lived subduction of an oceanic plate beneath a continental one ( Chen *et al.*, 2019), and can be divided into three segments, primarily based on variations in tectonic and geological factors along its length. The northern (NVZ), central (CVZ), southern (SVZ) and Austral (AVZ) volcanic zones, divided by magmatic activity interruptions, linked to ridge and spreading plate margins subduction and changes in the angles of subduction ( Barry *et al.*, 2022; Martinod *et al.*, 2010; Stern, 2004).

The CVZ, located between 16 °S and 28 °S, has intense volcanic activity, and features a high concentration of stratovolcanoes ( Stern, 2004), including some of the most active ones in South America, such as Lascar, Sabancaya and Ubinas ( Aguilera *et al.*, 2022; Stern, 2004; Stern *et al.*, 2007). It is characterised by a unique type of subduction, where angle of subduction of the subducting plate reaches less than 30 degrees in some sections ( Bartels *et al.*, 2022). The last Andean magmatic cycle, which is responsible for the last volcanic Arc, the current CVZ, started around 27 Ma (Late Oligocene), as a consequence of the Farallon plate break up, forming Cocos and Nazca plates. From north to south, the Andes taper from approximately 500 km across the Andean plateau to around 200 km at 35 °S, while crustal thickness decreases from over 65 km at 30 °S to less than 55 km further south ( Giambiagi *et al.*, 2022).

## Methods

### Sample selection and environmental parameters

**Figure 1.**
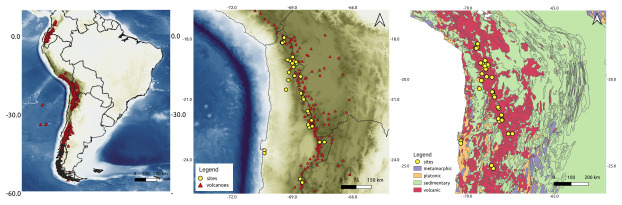
Map showing the location of the sampling sites and their geological context. **A**) General overview of the Andean Volcanic Zones. The sampled area presented in ( **B**) and ( **C**) is marked with a red rectangle; **B**) Topographic map showing the location of the sampled seeps; and **C**) geological maps of the sampled area showing the main geological units ( “Geological Map Of South America At a Scale of 1:5,000,000”, 2019), https://doi.org/10.32685/10.143.2019.929) (geological basemap from Gómez *et al.*, 2019).

**Figure 2.**
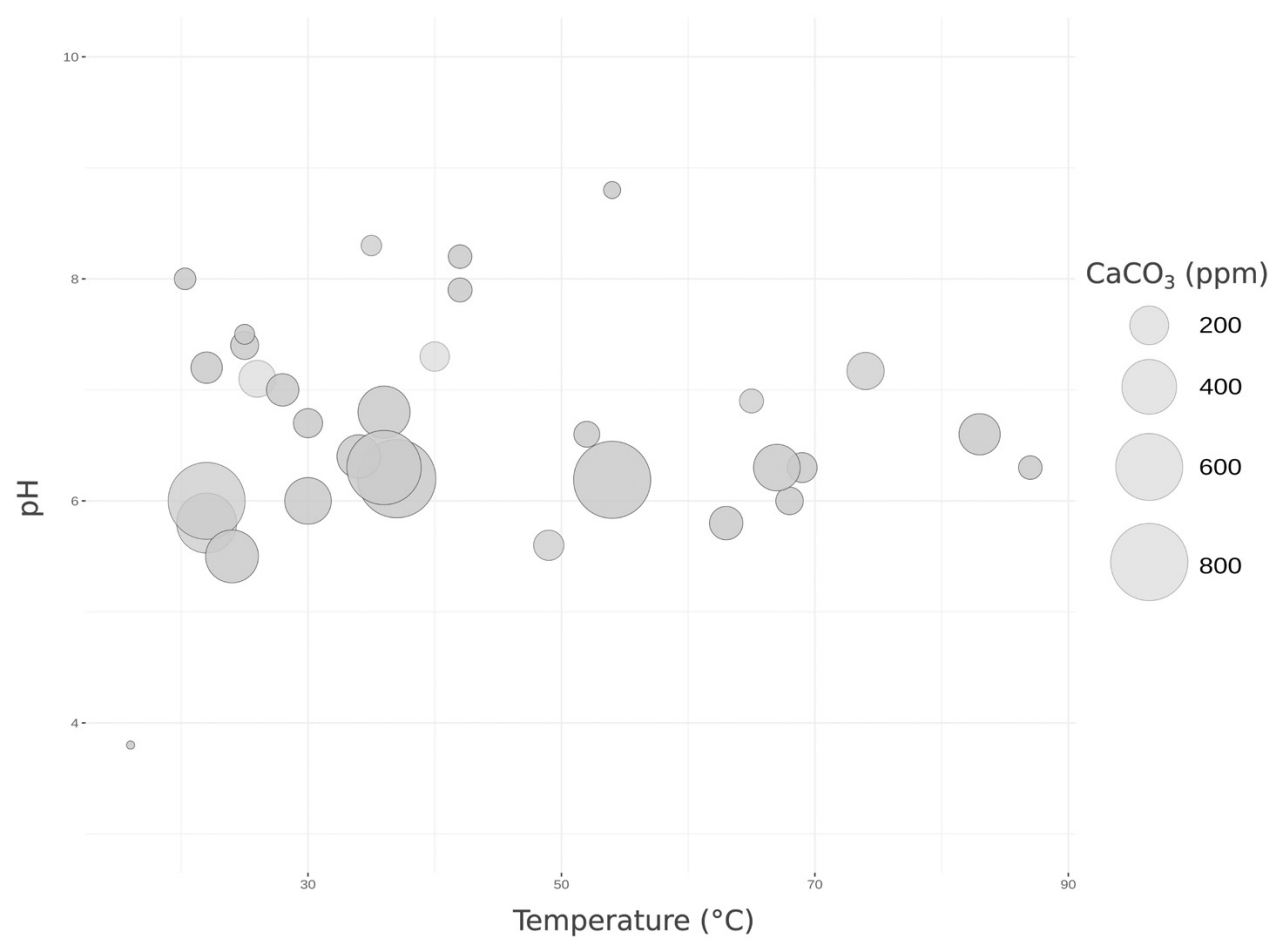
Graph showing the temperature, pH, and total alkalinity of the sampled locations. Temperature in °C and total alkalinity in ppm of CaCO _3 _(concentrations are proportional to the size of the circles).

We collected gases, fluids, and sediments from fumaroles and deeply-sourced seeps ( Giovannelli *et al.*, 2022b). In addition to including local scientists in our group and contacting national authorities to obtain permission for our work, we obtained permission from the individuals who owned the land on each site, only sampling after permission was granted. Using the sampling approach proposed by Fullerton *et al.* (2021), at each sampling site we identified the main water outlet by using a combination of field observation and measurements, to sample the pristine gases and fluids, minimising their interaction with the surface and the atmosphere. We also collected superficial sediment deposits that were constantly overwashed by the subsurface fluids, as well as background soil samples from the nearby area that did not show geothermal alterations. The rationale behind this type of sampling approach was to maximise the subsurface information that can be extrapolated from the data, knowing which communities are found exclusively in the seep and those shared with the surrounding areas ( Giovannelli *et al.*, 2022b). Each sampling location was photographed using a FLIR C2 thermal camera and a digital camera ( Figure 1). Total alkalinity (expressed as mg/L CaCO _3_), and silica were analysed in situ by (i) acidimetric titration using HCl 0.03 N, phenolphthalein and bromophenol blue as indicators, and (ii) molecular spectrophotometry (Hanna HI 96770C; accuracy: ±1 mg/L). Temperature, conductivity, pH, redox potential, total suspended solids and dissolved oxygen were measured in the field using a thermocouple and a multiparametric probe (HANNA, HI98196, accuracy: ±0.15 °C, ±0.02 pH) ( Figure 2). Water emission rates were estimated by measuring the spring outlet dimensions and the water flow velocity using a FL-K1 stream flow metre, handheld rod with impeller and LCD readout (JDC Instruments Electronics Flowatch Flowmeter). Gas fluxes were estimated by placing a volume-calibrated inverted funnel full of water over the bubbling source and measuring the time taken to displace a known volume of water.

### Geochemistry samples acquisition

***Dissolved gas geochemistry.*** Gas phase samples and water samples for dissolved gases were collected in pre-evacuated 250 ml Giggenbach bottles containing 50 ml of 4 N NaOH minimising atmospheric contamination ( Barry *et al.*, 2019). Copper tubes for the determination of the noble gas composition were collected at each site. Gases and fluids were first flushed into silicone tubes and through 3/8-inch copper tubes, that were then closed with stainless steel clamps after extensive flushing, trapping the sample inside the copper tubing ( Barry *et al.*, 2022). The tubing used for Cu-tubes was also used to collect gas samples using Giggenbach bottles.

***Aqueous and sediment geochemistry.*** At each site, we took sediment samples as close as possible to the water outlet point, and collected them into 50 ml conical tubes, prewashed in soap and water and then in acid, to avoid any possible metal contamination. We collected two 50 ml conical tubes of 0.22 µm filtered fluids for ion chromatography (IC) and ion coupled plasma mass spectrometer (ICP-MS) analysis ( Correggia *et al.*, 2023; Correggia *et al.*, 2024). Samples for ICP-MS analysis were acidified with nitric acid to estimate acid-soluble metals. We also collected a 10 ml anoxic vial for dissolved inorganic carbon. All the equipment was acid washed to avoid metal contamination.

### Biological samples acquisition

We sampled sediments and fluids for molecular analysis, with the sampling approach proposed by Fullerton *et al.*, 2021. For each site, we filtered between 2 to 4 litres of hydrothermal fluids through a Sterivex 0.22 µm filter (Millipore Sigma) using a portable 3D printed peristaltic pump powered through an battery portable drill. The plans to print and reproduce the peristaltic pump are available at DOI: http://dx.doi.org/10.5281/zenodo.12742933. The fluids were collected as close as possible to the venting outlet to prevent mixing with the surface as described previously ( Giovannelli *et al.*, 2022b). Using 50 ml falcon tubes, we collected sediments as close as possible to the water outlet, and background soils choosing the closest point to the seep which showed no signs of influence from the hydrothermal activity. Filters and sediments were immediately frozen onsite at liquid nitrogen temperature in a cryogenic dry shipper (ThermoFisher Scientific, Arctic Express 20). To identify and count cells using flow cytometry, we sampled a 2 ml cryovial of fluid and a 2 ml cryovial of sediment, both of which were preserved in 3 % paraformaldehyde. Finally, we filled 100 ml vials with biofilms of various colours, microbial mats, fluids, and sediments, for culturing and isolation purposes.

## Sites and samples log

We named each site using the following convention:

- The ISO Alpha-3 country code or a two-letter project specific code is used to assign the name to the expedition followed by a two numeric digit indicating the year called ExpID (e.g., MNG23);
- Each sampling site is identified using a two-letter code selected from the name of the location, called SiteID (e.g., NR for …);
- Each sampling effort is identified by the SiteID followed by the sampling date in the YYMMDD format, called a CollectionID (e.g., NR230731). This gives a unique identifier of the sample based on the location and the date;
- The different samples collected during each collection are identified by each participating group using tags specific for the type of analysis carried out.

Physicochemical characteristics of each site and the measured parameters in the field are reported in Table 1. The description of each sampled location follows together with pictures useful for future identification of the site. A .kmz file containing the exact location of the sampled sites is available on the online GitHub repository associated with this report ( https://github.com/giovannellilab/Chile_2022_expedition.git).

**Table 1.** Sampled sites with main environmental and physicochemical characteristics.

SiteID	CollectionID	Temp (°C)	pH	Latitude (°N)	Longitude (°E)	Altitude (m)	SPC (μS/cm)	Alkalinity (ppm CaCO3)	Water flux	Gas flux
LC220316	LC	40	7.3	-23.5629	-70.4002	13	15170	114.1	9.52	0.000
QN220317	QN	26	7.1	-23.6979	-70.4064	94	39800	180.2	0.44	0.000
CH220319	CH	22	5.8	-22.4173	-68.1726	3754	8476	480.4	0.32	0.103
RS220319	RS	22	6.0	-22.2781	-68.2277	3084	9108	792	1.89	2.000
AL220320	AL	83	NA	-23.1459	-67.6553	4747	NA	NA	0.00	NA
AV220320	AV	68	6.0	-23.1492	-67.6585	4705	5332	99	1.26	0.246
LN220321	LN	52	6.6	-23.1468	-67.4192	4227	52600	87.1	19.28	0.003
ET220322	ET	87	6.3	-22.3307	-68.0118	4278	25900	72.1	NA	NA
GB220322	GB	83	6.6	-22.3570	-68.0226	4287	8710	225.2	5.93	0.802
CA220323	CA	25	7.4	-22.0650	-68.0592	4061	277	102.1	17.64	0.000
OL220324	OL	147	NA	-20.9412	-68.4833	5312	NA	NA	NA	NA
CC220324	CC	20.3	8	-21.0251	-68.4508	4266	91	60.1	32.50	0.000
CR220324	CR	42	7.9	-22.0650	-68.0593	4125	3541	75.1	10.32	0.000
OA220325	OA	28	7.0	-21.6879	-68.2149	3736	3630	138.1	9.24	NA
VA220325	VA	22	7.2	-21.6095	-68.2501	3795	7360	129.1	2.46	0.000
TM220326	TM	54	8.8	-20.0704	-69.2118	2804	1120	37	12.02	0.000
CN220327	CN	37	6.2	-19.8854	-68.6013	3906	10120	819.7	7.56	0.326
TL220327	TL	69	6.3	-19.8518	-68.9065	3999	3000	117.1	12.53	0.070
IR220328	IR	408	NA	-20.7345	-68.5574	4977	NA	NA	NA	NA
IS220328	IS	38	2.4	-20.7259	-68.5862	4042	21120	NA	1.68	0.000
SR220328	SR	35	8.3	-20.5405	-69.3260	1322	8089	54	0.67	0.000
CZ220329	CZ	42	8.2	-19.6835	-69.1772	3423	679	72.1	272.61	0.000
PZ220330	PZ	74	7.17	-19.4085	-68.9585	4205	16450	183.2	2.52	0.004
PD220330	PD	86	NA	-19.4128	-68.9579	4222	NA	NA	NA	NA
PJ220330	PJ	30	6.7	-19.1208	-68.9098	4242	1769	111.1	1.86	0.000
EQ220330	EQ	30	6.0	-19.2351	-68.7920	3901	2500	288.3	136.08	0.000
IV220331	IV	97	NA	-19.1622	-68.8349	5163	NA	NA	NA	NA
TT220331	TT	67	6.3	-19.1134	-69.1384	4067	20610	288.3	0.72	0.000
LA220331	LA	36	6.8	-19.0588	-69.2528	3728	20880	360.3	7.12	NA
LR220331	LR	24	5.5	-19.8518	-68.9061	4079	29460	372.3	110.76	0.000
LV220331	LV	49	5.6	-19.0570	-69.2534	3731	28910	120.1	10.92	0.000
LP220401	LP	25	7.5	-19.2330	-69.0104	4157	582	51	13.32	NA
PQ220401	PQ	63	5.8	-18.9132	-68.9992	4283	12200	147.1	37.83	NA
CP220402	CP	16	3.8	-17.9501	-69.4362	4151	1620	8	1.33	0.000
CE220402	CE	54	6.19	-17.9555	-69.4236	4141	34400.0	795.7	4.66	0.035
CU220402	CU	34	6.4	-18.1700	-69.4309	4473	2610	249.2	6.66	0.005
PR220403	PR	36	6.3	-18.1979	-69.5385	3785	25410	738.6	0.10	0.008
JR220403	JR	65	6.9	-18.2102	-69.5105	4050	6129	75.1	13.00	0.000

### Site 1 - La Chimba, LC220316 (-23.5629 °N, -70.4002 °E)

**Figure 3.**
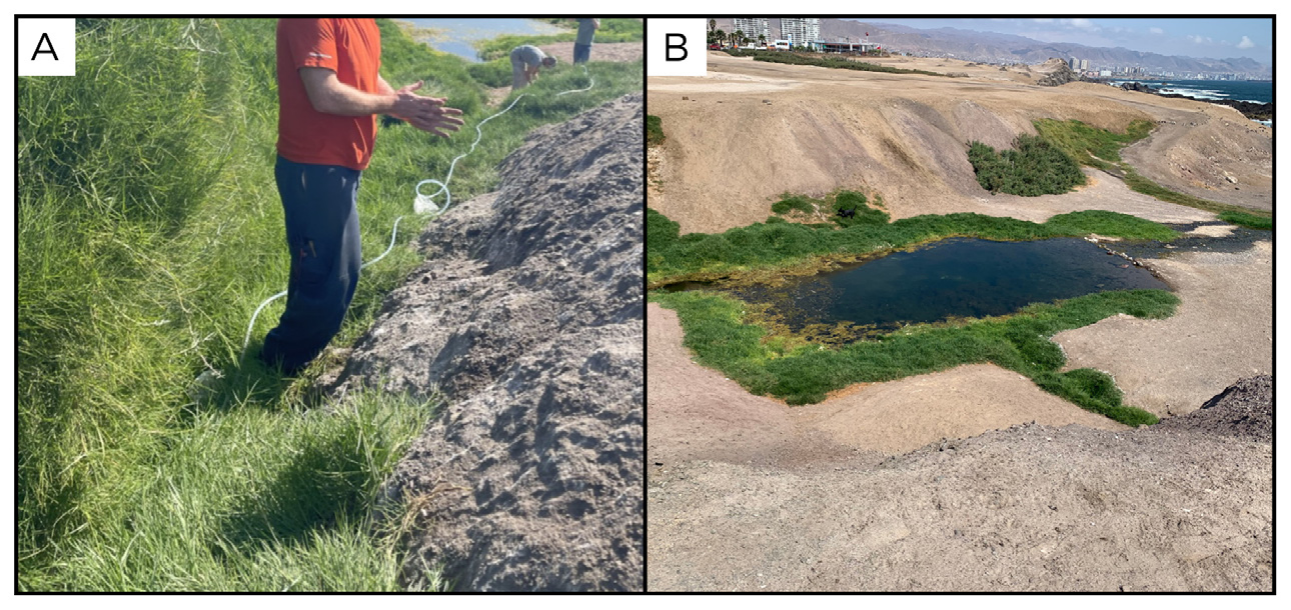
La Chimba, LC220316. Fluids were sampled from a small water outlet point between the grass. **A**. Detail of the sampling location where the fluids were collected. **B**. Large view of the sampling site. Photograph taken by author DB for this publication.

The LC220316 ( Figure 3) spring was the first site sampled during this expedition. It is situated approximately 30 metres from the seashore, near the city of Antofagasta, at an elevation of 13 metres above sea level. The site features a water outlet hidden within the grass ( Figure 3), which feeds a small pool at its base. No gas bubbles are present. The spring and its associated pool are encompassed by various forms of vegetation. The site is surrounded by evidence of animal activity and traces, and human-generated litter.

### Site 2 - Quebrada Negra, QN220317 (-23.6979 °N, -70.4064 °E)

**Figure 4.**
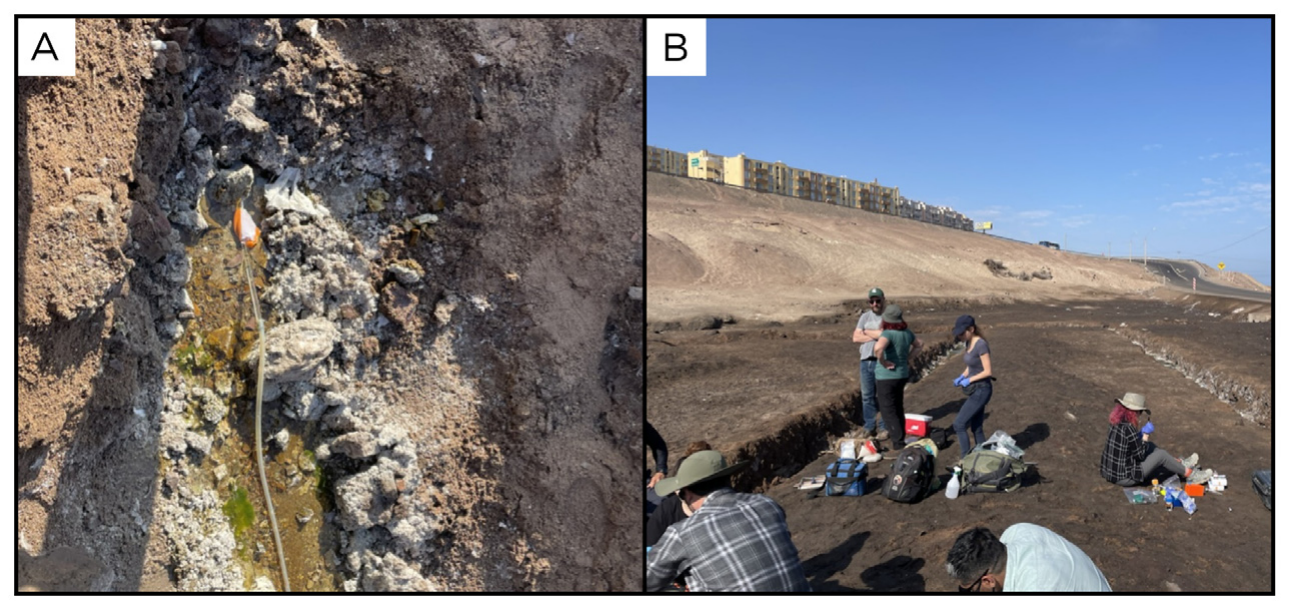
Quebrada Negra, QN220317. **A**. Detail of the sampling location where the fluids were collected. **B**. Large view of the sampling site. Photograph taken by author DB for this publication.

Site QN220317 lies on the edge of the city of Antofagasta, within the dry bed of an arheic basin, at an altitude of 93 metres above sea level ( Figure 4). The water outlet was identified inside a channel into the ground. The site is located about 500 metres from the road, so it is easily accessible by car. The site harbours indications of human impact, as remnants of discarded trash are found within it, alongside signs of animal presence such as tracks and traces.

### Site 3 - Chitor, CH220319 (-22.4173 °N, -68.1726 °E)

**Figure 5.**
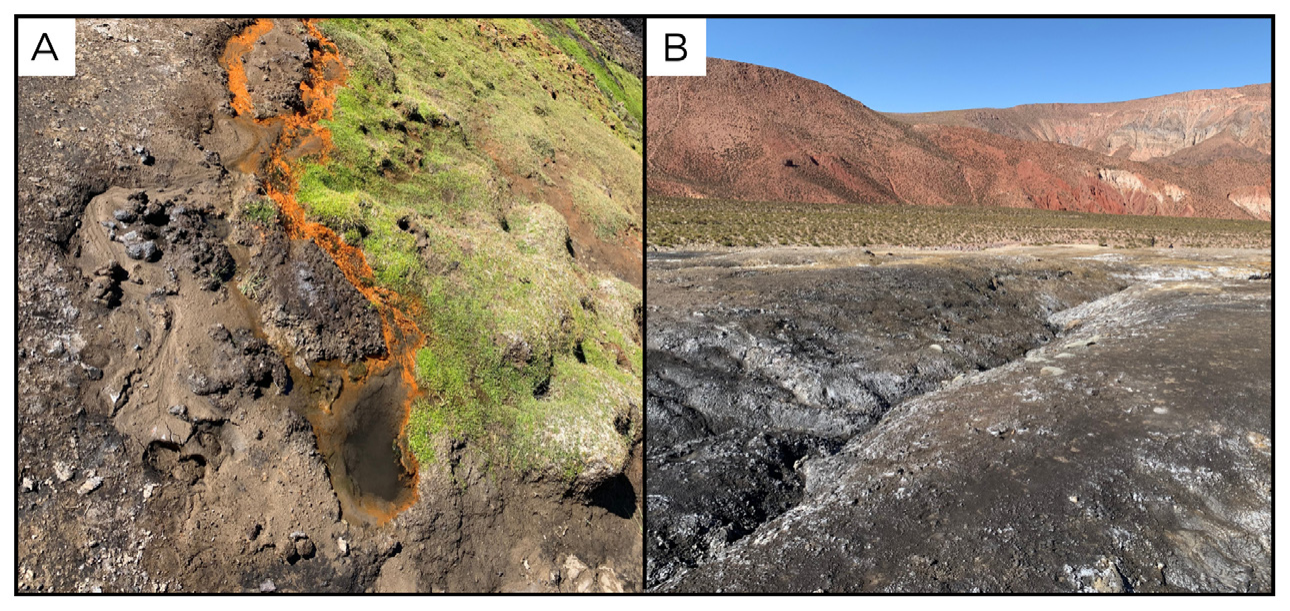
Chitor, CH220319. **A**. Detail of the sampling location where the fluids were collected. **B**. Large view of the sampling site. Photograph taken by author DB for this publication.

Site CH220319 is nestled in the heart of the Atacama Desert, approximately 90 kilometres from the city of Calama ( Figure 5). This remote location is at an elevation of 3754 metres above sea level. Chitor is about 150 metres away from the main road, and accessible by car. The site is located in a flat basin surrounded by mountains and hills, suggesting that rainwater likely funnels into this basin. The water leak point emerges in an area surrounded by sparse vegetation, with the presence of animal tracks. Around the water outlet there is evidence of iron precipitation, and the mound has some carbonate deposition. The water flow is low and CO _2_-rich.

### Site 4 - Rio Salado, RS220319 (-22.2781 °N, -68.2277 °E)

**Figure 6.**
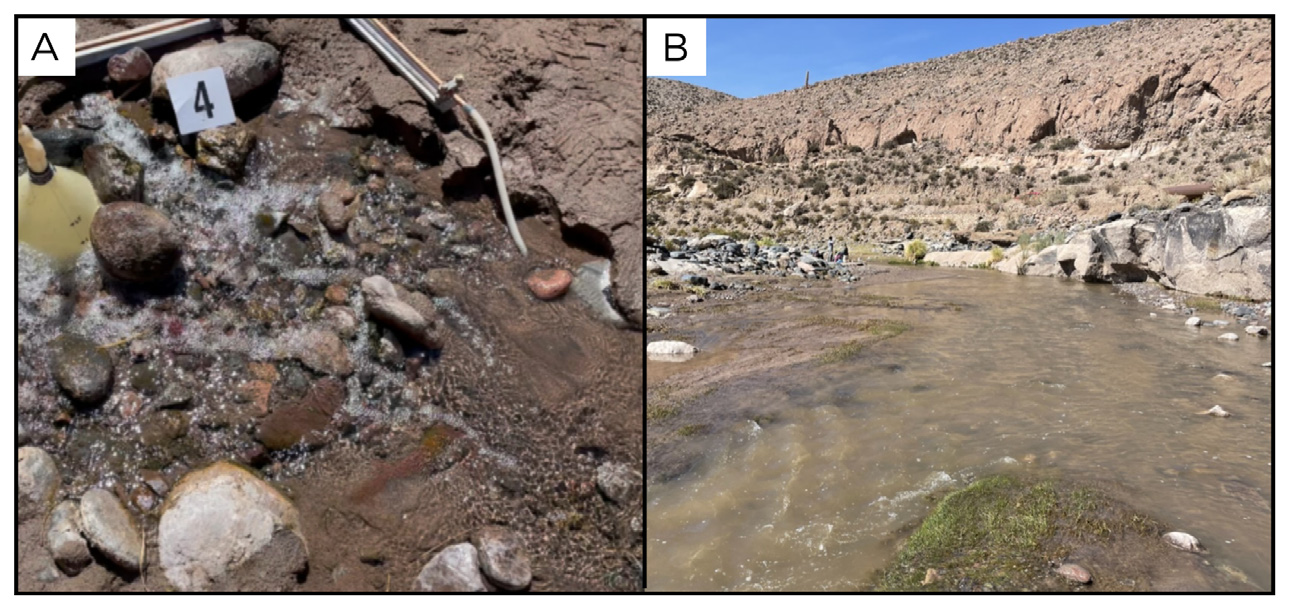
Rio Salado, RS220319. Fluids were sampled from a bubbling water outlet point next to the river. **A**. Detail of the sampling location where the fluids were collected. **B**. Large view of the sampling site. Photograph taken by author DB for this publication.

Site RS220319 was sampled on the bank of the Rio Salado river, that flows near the city of Calama, at an altitude of 3084 metres above sea level ( Figure 6). The site is close to a road, and we crossed the river on foot to reach the site we selected for sampling. On the river bank there are three water outlet points, all close to each other and each with high flux of gas bubbles.

### Site 5 - Alitar fumaroles, AL220320 (-23.1459 °N, -67.6553 °E)

**Figure 7.**
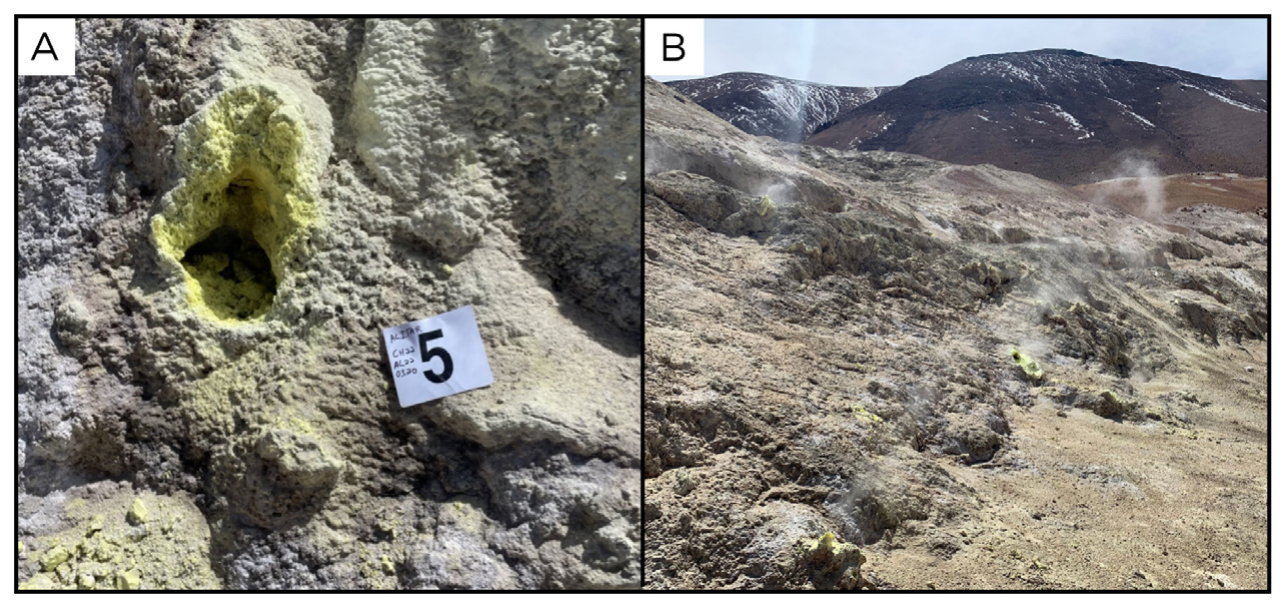
Alitar fumaroles, AL220320. **A**. Detail of the sampling location where the samples were collected. **B**. Large view of the sampling site. Photograph taken by author DG for this publication.

Site AL220320 is located within the fumarole of the Alitar volcano, at an altitude of 4747 metres above sea level ( Figure 7). The site is located inside the volcano's crater, with diffuse degassing of H _2_S. Given the high temperature of the fumarole, we only collected sediments and gas samples at this site, and targeted an area with relatively lower temperature, 83°C for microbiological sampling.

### Site 6 - Alitar verde, AV220320 (-23.1492 °N, -67.6585 °E)

**Figure 8.**
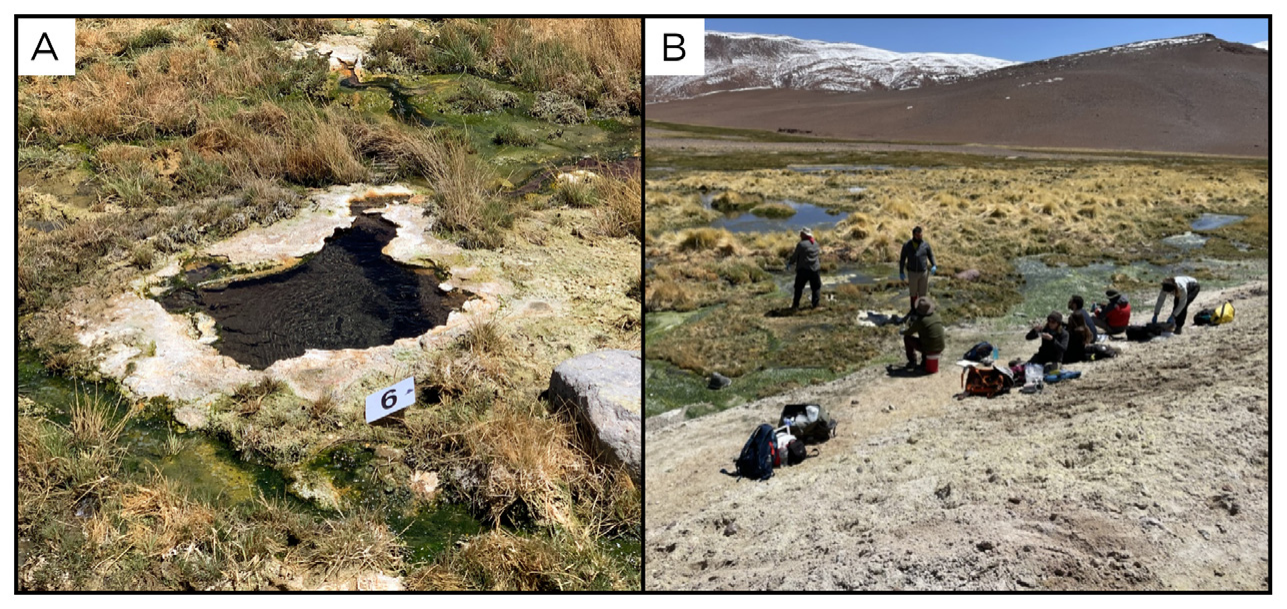
Alitar verde, AV220320. **A**. Detail of the sampling location where the fluids were collected. **B**. Large view of the sampling site. Photograph taken by author JP for this publication.

Site AV220320 is located near the Alitar fumaroles site, at an altitude of 4705 metres above sea level ( Figure 8). It is a marshy area fed by emissions of hot underground water, at around 68 ° C, with moderate bubbling. The pool is surrounded by limestone encrustations, and while the inside of the sampled pool is clear and free from vegetation and algae, all around there is vegetation, animal tracks and green and white biofilm.

### Site 7 - Laguna Negra, LN220321 (-23.1468 °N, -67.4192 °E)

**Figure 9.**
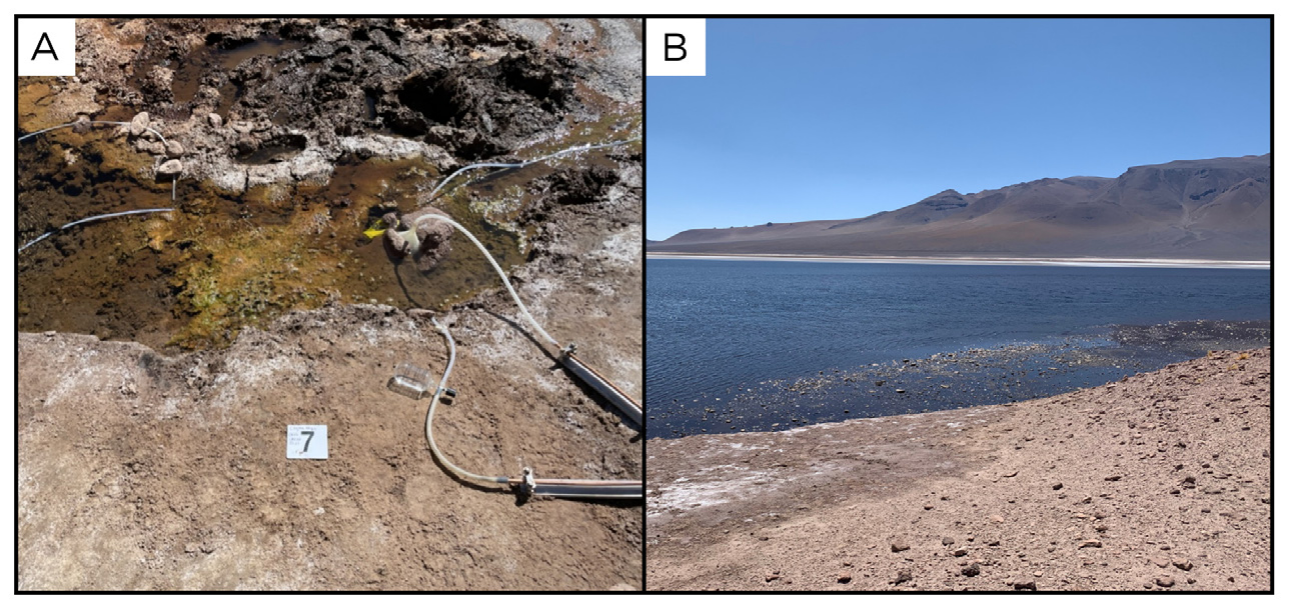
Laguna Negra, LN220321. Fluids were sampled from a water outlet point near the lake. **A**. Detail of the sampling location where the fluids were collected. **B**. Large view of the sampling site. Photograph taken by author DG for this publication.

Site LN220321 is near the shore of a system of three salares and lagoons, alternating between periods of desiccation and replenishment by rainwater cascading from the surrounding hills ( Figure 9). This site is easily accessible by car, and the water outlet point selected for the sampling is a 52°C water release along the shoreline of the Laguna Negra lagoon. The site has small, intermittent gas emissions, and inside the pool there are green and brown biofilms, surrounded by whitish carbonate concretions.

### Site 8 - El Tatio, ET220322 (-22.3307 °N, -68.0118 °E)

**Figure 10.**
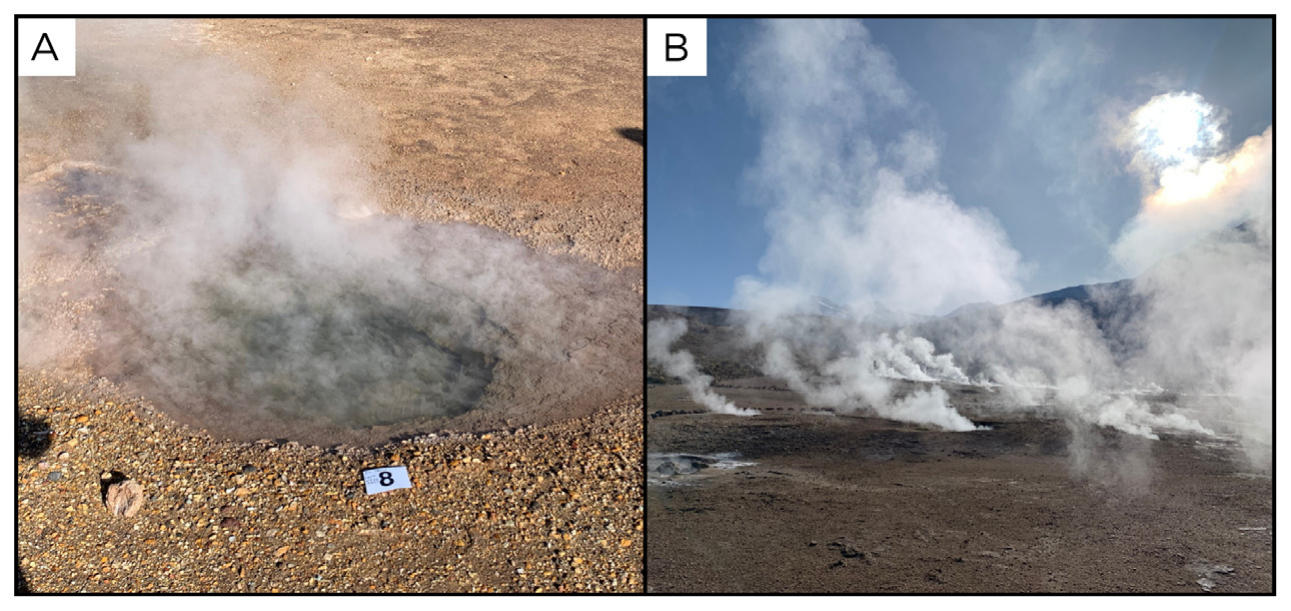
El Tatio, ET220322. Fluids were sampled from the pool, as close as possible to the water outlet. **A**. Detail of the sampling location where the fluids were collected. **B**. Large view of the sampling site. Photograph taken by author DG for this publication.

Site ET220322 is located within the El Tatio geothermal field, near San Pedro de Atacama ( Figure 10). With an altitude of 4278 metres above sea level, El Tatio is one of the highest-altitude geothermal fields in the world, known for its numerous geysers, hot springs, and fumaroles. Among the many pools, we chose to sample one with a temperature of 87°C, which was slightly lower than other pools. The sampled pool was surrounded by sandy and gravelly sediment, while inside the pool there was very little fine sediment, and mostly pieces of sinter encrustations that had fallen from the sides.

### Site 9 - Geyser Blanco, GB220322 (-22.3570 °N, -68.0226 °E)

**Figure 11.**
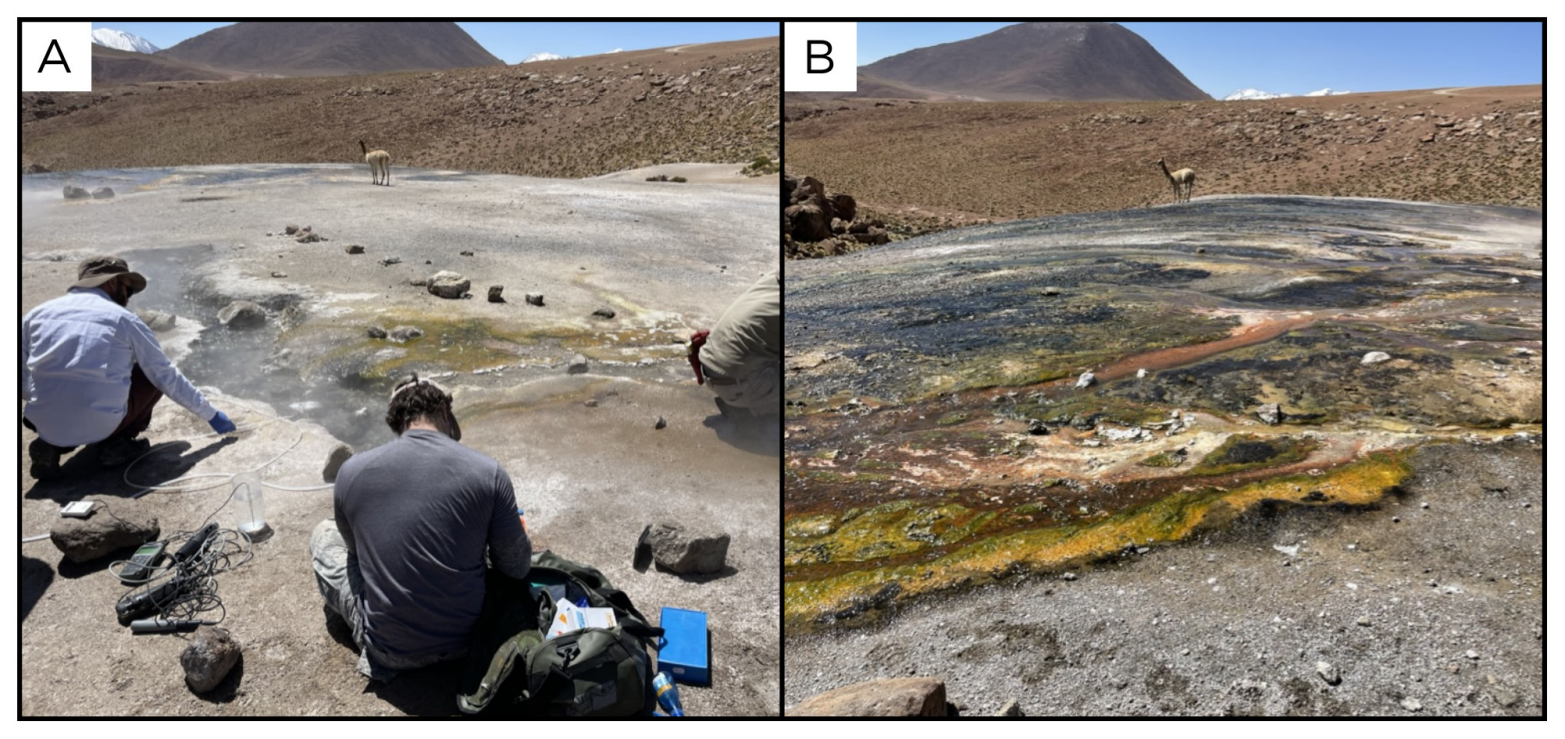
Geyser Blanco, GB220322. **A**. Detail of the sampling location where the fluids were collected. **B**. Large view of the sampling site. Photograph taken by author DB for this publication.

Site GB220322 is approximately 3 km from the El Tatio geothermal field, with an altitude of 4287 metres above sea level ( Figure 11). It is a water emission site at 83°C, where water flows along a hill producing a small stream with limestone deposits as well as biofilms of various colours, including green, white and orange. The site lacked vegetation, but showed animal traces.

### Site 10 - Cabana, CA220323 (-22.0650 °N, -68.0592 °E)

**Figure 12.**
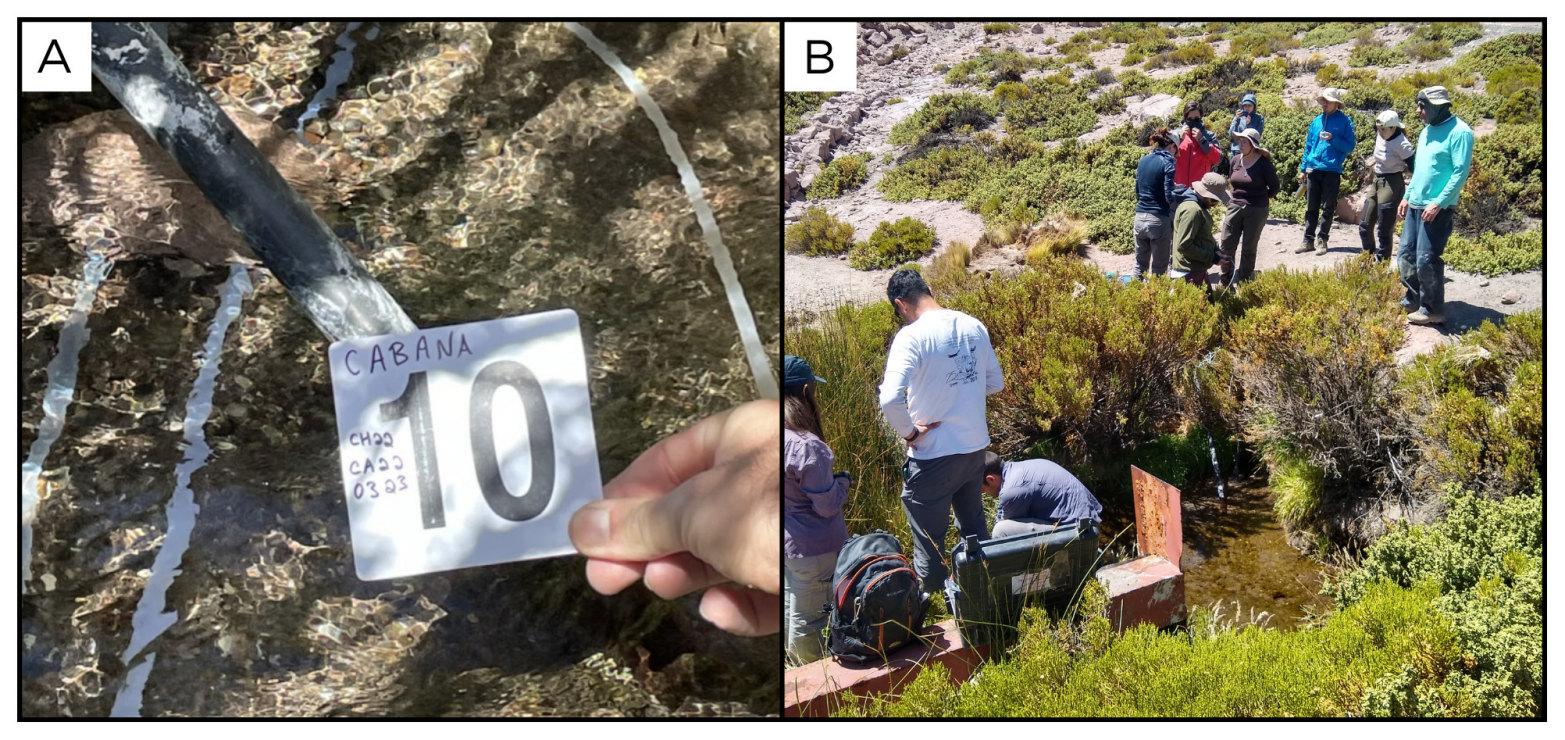
Cabana, CA220323. Fluids were sampled from the water outlet point, at the bottom of the pool. **A**. Detail of the sampling location where the fluids were collected. **B**. Large view of the sampling site. Photograph taken by author DG for this publication.

Site CA220323 is located at about 5 km from Cabana at an altitude of 4061 metres above sea level ( Figure 12). It is a hot spring surrounded by vegetation, located in a valley surrounded by hills and mountains. In the pool, the high water flow is artificially channelled through a series of pipes. The water flows out at a temperature of 25°C. There were fish inside the pool. The site is close to old sulphur mine tailings and buildings distributed in the area.

### Site 11 - Olca Volcano, OL220324 (-20.9412 °N, -68.4833 °E)

**Figure 13.**
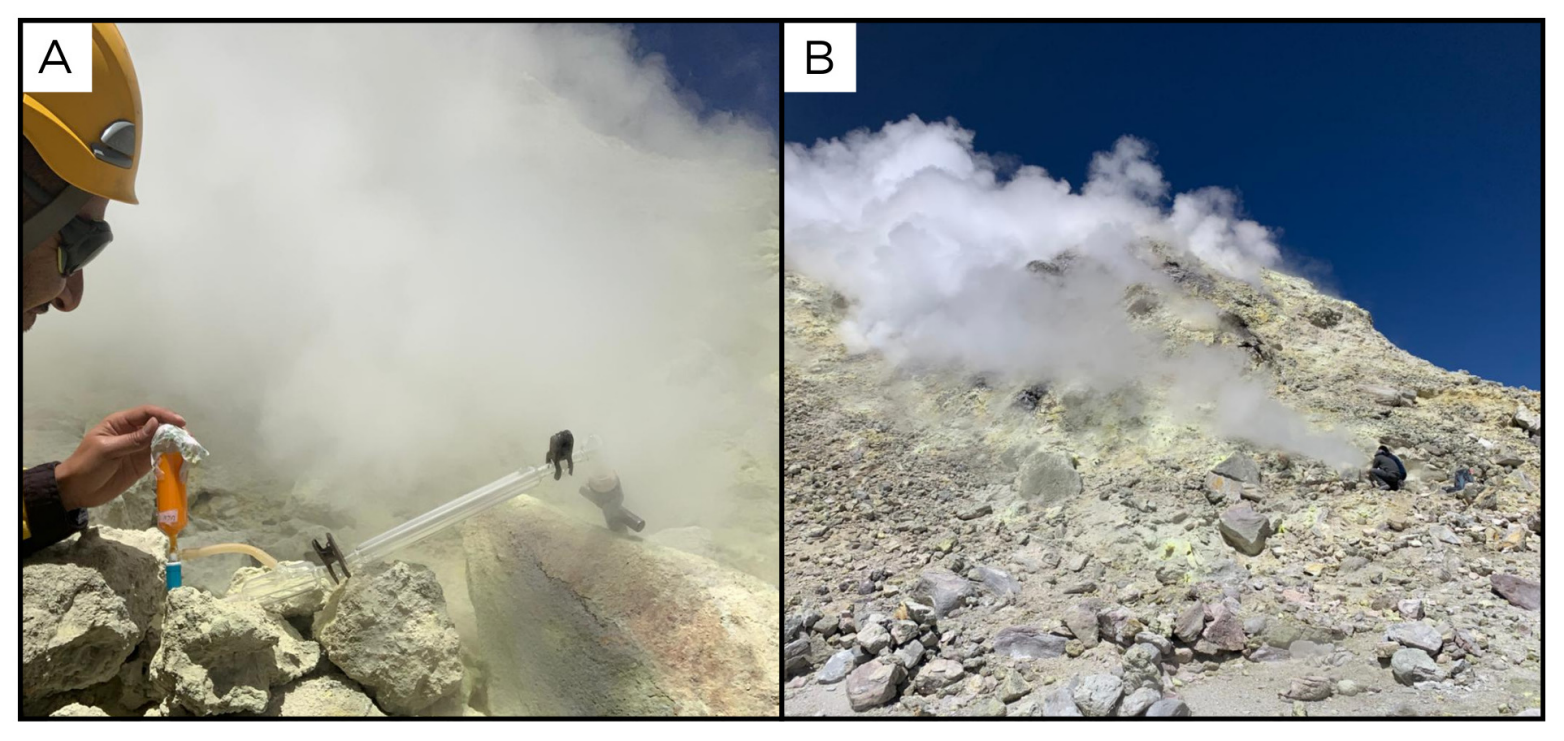
Olca Volcano, OL220324. No fluids were sampled at this site. **A**. Detail of the sampling location. **B**. Large view of the sampling site. Photograph taken by author JP for this publication.

Site OL220324 is a fumarole field on the Olca volcano, at 5312 metres above sea level ( Figure 13). Olca is a stratovolcano located on the border with Bolivia, and its gases emissions come from a fumarole field over the crater's dome. The temperature recorded at the sampling point is 147°C, and no fluids for biological analysis were sampled.

### Site 12 - Vega Churchilla, CC220324 (-21.0251 °N, -68.4508 °E)

**Figure 14.**
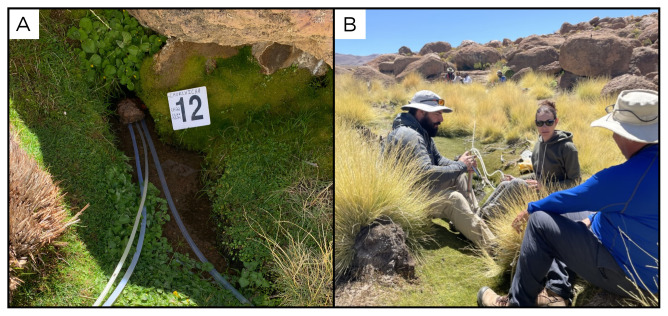
Vega Churchilla, CC220324. Fluids were sampled from the water outlet point. **A**. Detail of the sampling location where the fluids were collected. **B**. Large view of the sampling site. Photograph taken by author DB for this publication.

Site CC220324 is located in the municipality of Ollagüe, a small city at the base of the andesitic Ollagüe stratovolcano ( Figure 14). The point of emission of the water, which comes out at a temperature of 20.3 °C, is located within a relatively flat area, surrounded by hills and mountains. The entire area is covered by low vegetation and ignimbrites outcrops. The site is at an altitude of 4266 metres above sea level, and has no gas phase.

### Site 13 - Carcote, CR220324 (-22.0650 °N, -68.0593 °E)

**Figure 15.**
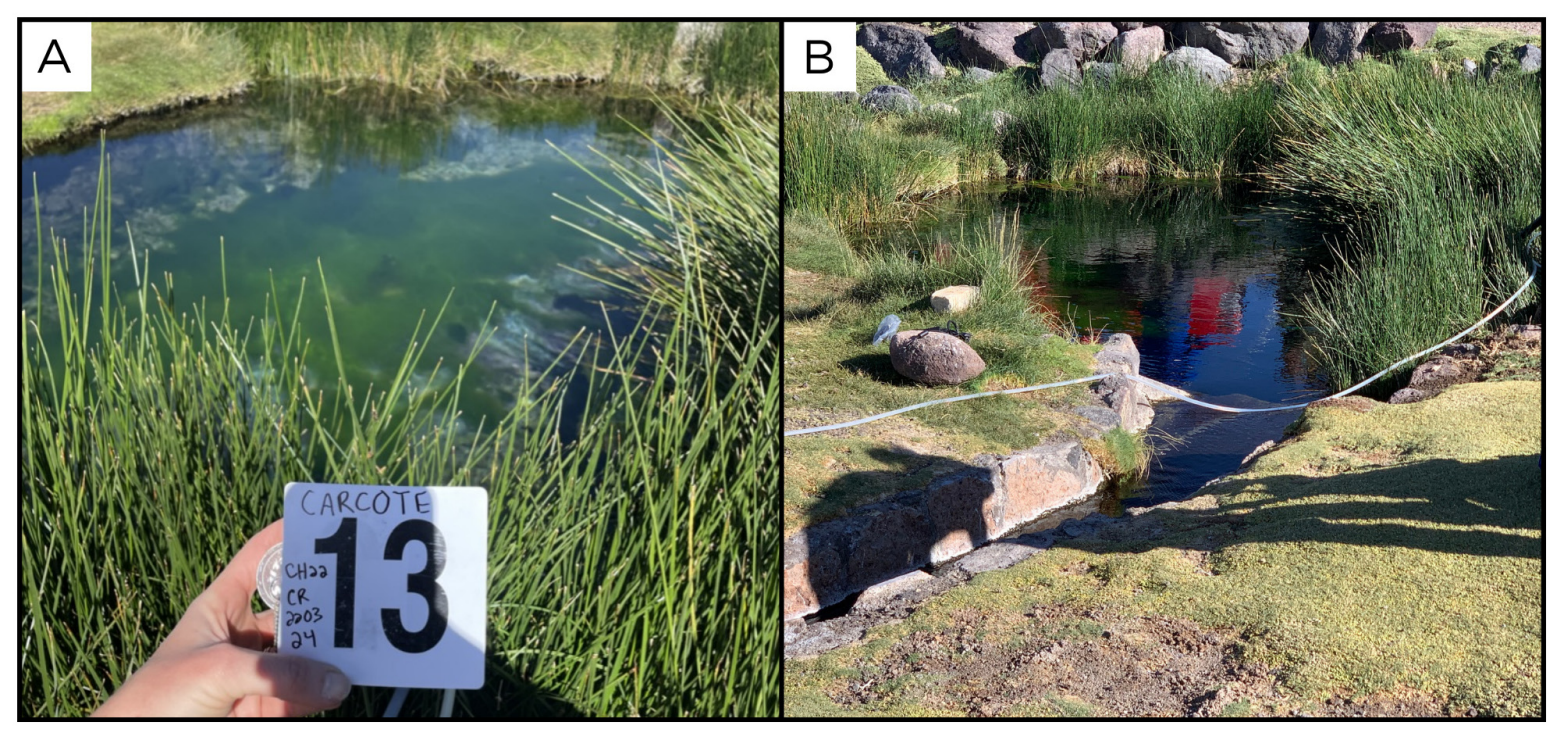
Carcote, CR220324. Fluids were sampled from the water outlet point, before reaching the pool. **A**. Detail of the pool. **B**. Large view of the sampling site. Photograph taken by author KGL for this publication.

Site CR220324 is located 4125 metres above sea level, near the Salar de Carcote ( Figure 15). The site consists of a water outlet at 42°C, with no gas, feeding a pool used by tourists.

### Site 14 - Ojo de Ascotán vertiente, OA220325 (-21.6879 °N, -68.2149 °E)

**Figure 16.**
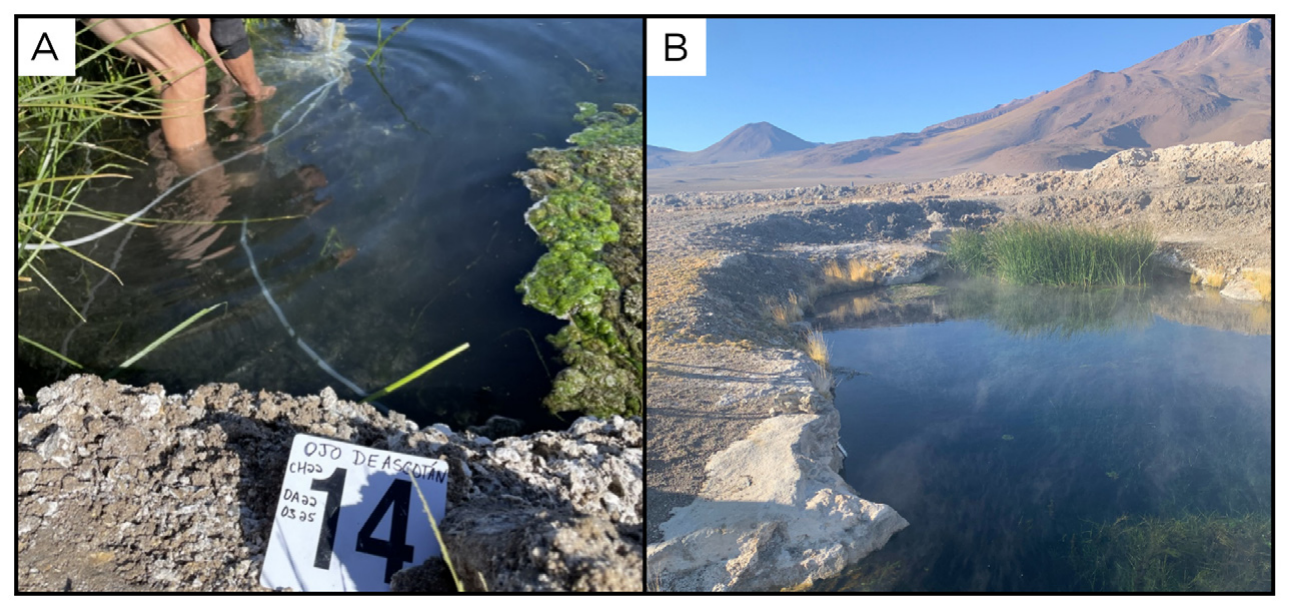
Ojo de Ascotán vertiente, OA220325. Fluids were sampled from the water outlet point, at the bottom of the pool. **A**. Detail of the sampling location where the fluids were collected. **B**. Large view of the sampling site. Photograph taken by author DB for this publication.

Site OA220325 has an altitude of 3736 metres above sea level, and is one of a series of pools in the middle of a salar ( Figure 16). Fluids and bubbles come out of a horizontal pipe inside the pool sampled. The source of the water, which we measured at 28 ° C, is apparently a 200 m deep well in the Salar de Ascotán, as told to us by the caretakers of the site.

### Site 15 - Vertiente 10 Ascotán, VA220325 (-21.6879 °N, -68.2149 °E)

**Figure 17.**
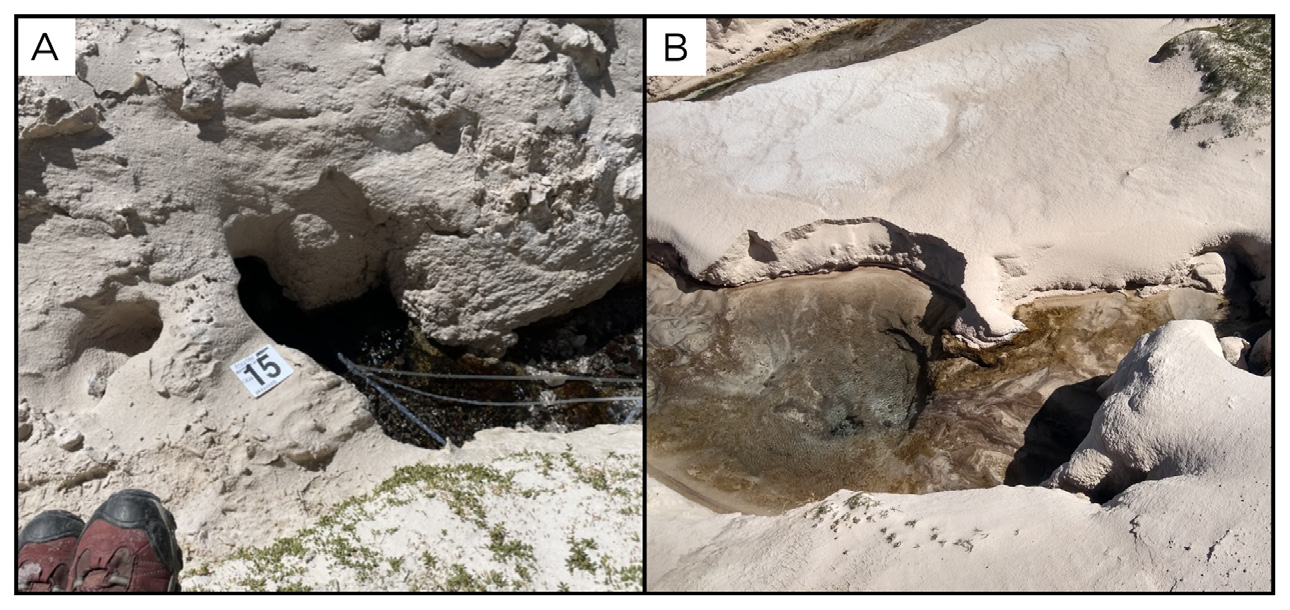
Vertiente 10 Ascotán, VA220325. Fluids were sampled from the water outlet point, at the bottom of the pool. **A**. Detail of the sampling location where the fluids were collected. **B**. Large view of the sampling site. Photograph taken by author KGL for this publication.

Site VA220325 is a salty spring on the edge of the Salar de Ascotán, 3795 metres above sea level and water is 22 ° C ( Figure 17). The water flows out of sandy sediment and feeds a small pool with algae, aquatic vegetation, and animals.

### Site 16 - Termas de Mamiña, TM220326 (-20.0704 °N, -69.2118 °E)

**Figure 18.**
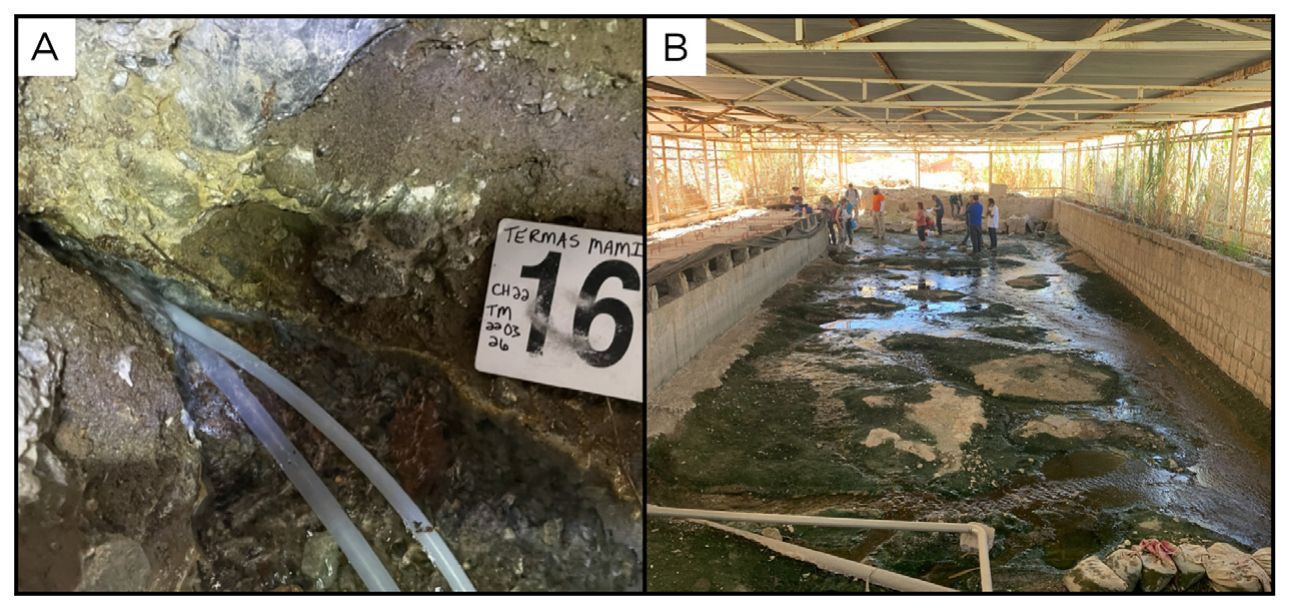
Termas de Mamiña, TM220326. Fluids were sampled from the water outlet point. **A**. Detail of the sampling location where the fluids were collected. **B**. Large view of the sampling site. Photograph taken by author KGL for this publication.

Site TM220326 is a spring in Mamiña, a small village 130 kilometres east of Iquique, 2804 metres above sea level ( Figure 18). Fluids were 54 ° C, and with a pH of 8.8, making this the most alkaline spring sampled. The water flows into an artificial structure, with extensive mats and biofilms of different colours, and eventually is used by the inhabitants of the nearby town.

### Site 17 - Termas de Cancosa, CN220327 (-19.8854 °N, -68.6013 °E)

**Figure 19.**
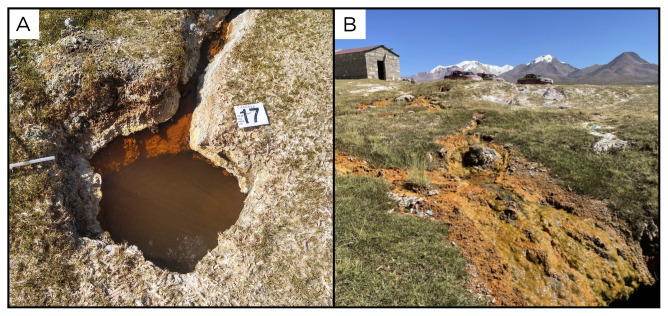
Termas de Cancosa, CN220327. Fluids were sampled from the water outlet point, at the bottom of the pool. **A**. Detail of the sampling location where the fluids were collected. **B**. Large view of the sampling site. Photograph taken by author DB for this publication.

Site CN220327 is 3906 metres above sea level and 37 ° C, with strong bubbling, iron precipitation and travertine deposition all around it ( Figure 19). At approximately 50 metres from the spring there is a small stone hut with a pool inside, showing strong bubbling.

### Site 18 - Termas de Lirima, TL220327 (-19.8518 °N, -68.9065 °E)

**Figure 20.**
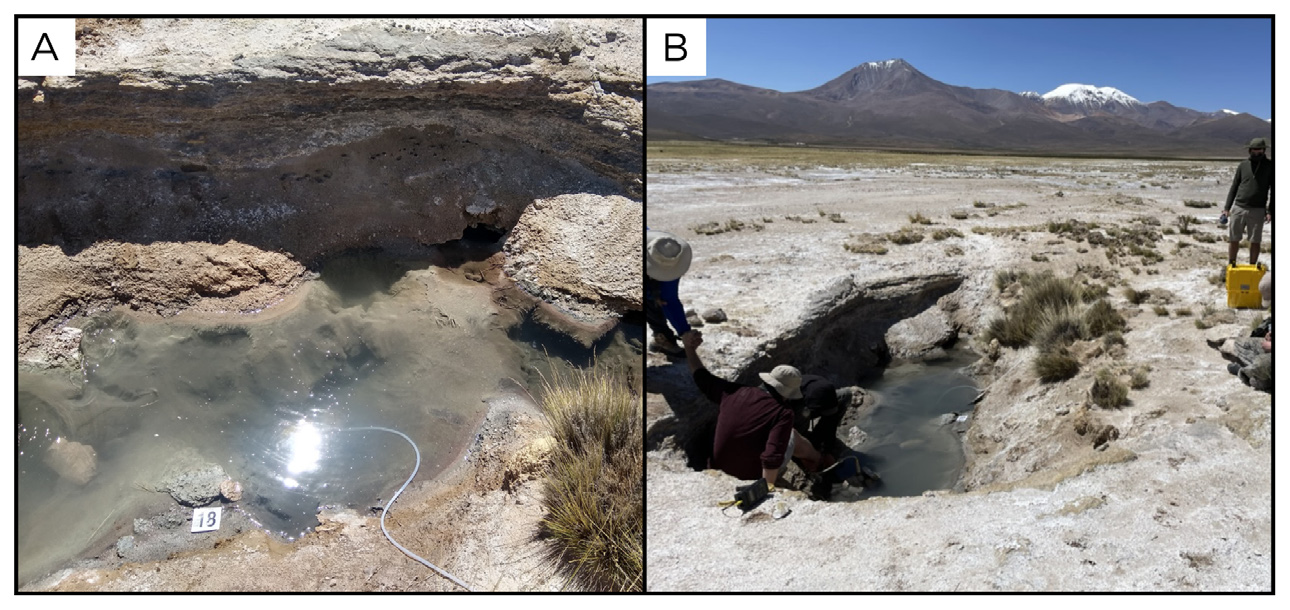
Termas de Lirima, TL220327. Fluids were sampled from the water outlet point, at the bottom of the pool. **A**. Detail of the sampling location where the fluids were collected. **B**. Large view of the sampling site. Photograph taken by author DB for this publication.

Site TL220327 is on a large sinter dome, with bluish coloured pools, all characterised by strong and intermittent bubbling ( Figure 20). The site is located at an altitude of 3999 metres above sea level, and the fluids are 69 ° C. The pools are all surrounded by sinter concretions, inside them there is very fine and dark sediment, and around them are animal carcasses.

### Site 19 - Irruputuncu fumaroles, IR220328 (-20.7345 °N, -68.5574 °E)

**Figure 21.**
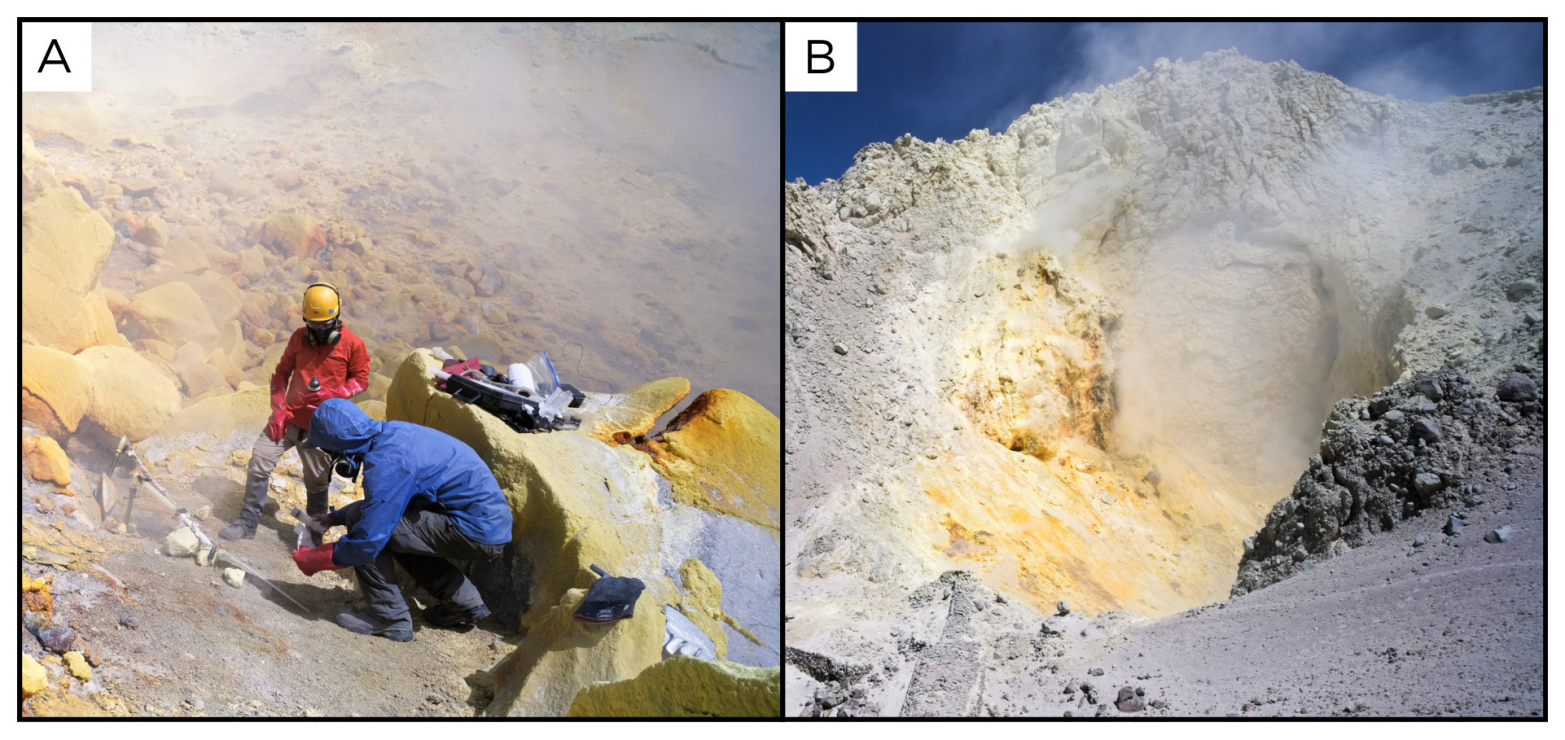
Irruputuncu fumaroles, IR220328. No fluids were available to be sampled at this location. **A**. Detail of the sampling location. **B**. Large view of the sampling site. Photograph taken by author JP for this publication.

Site IR220328 is a fumarole in the southernmost crater of the Irruputuncu volcano, at 4977 metres above sea level. At 408 ° C, this was the highest temperature site sampled in the expedition. All around the site there is strong degassing, with native sulphur deposits and high temperature acid gases ( Figure 21). Near the site there is an old sulphur mining infrastructure. No aqueous phase samples were available for collection at this specific site.

### Site 20 - Irruputuncu acid spring, IS220328 (-20.7259 °N, -68.5862 °E)

**Figure 22.**
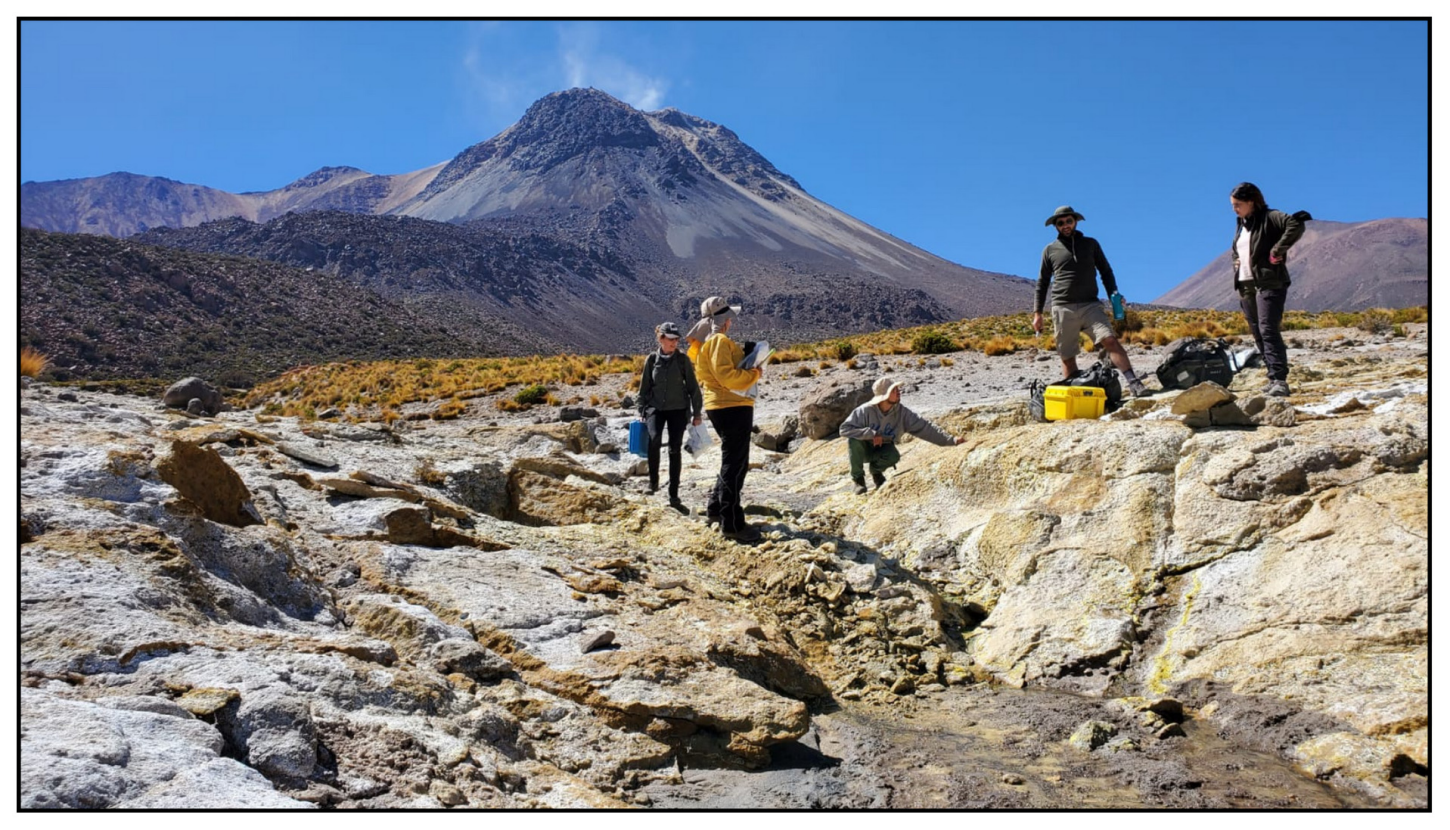
Irruputuncu acid spring, IS220328. Fluids were sampled from the water outlet point, near the pool. **A**. Detail of the sampling location where the fluids were collected. **B**. Large view of the sampling site. Photograph taken by author JP for this publication.

Site IS220328 is an acidic spring located near the base of the Irruputuncu volcano, at 4042 metres above sea level, and with a fluid temperature of 38 ° C ( Figure 22). With a pH of 2.4, it is the most acidic spring sampled during this expedition. There are two main water emissions at the sampling location, both with very very limited water flow. Only one of the two had sufficient flow to sample. Near the water outlet, the sediment is sparse and coarse.

### Site 21 - Santa Rosita, SR220328 (-20.5405 °N, -69.3260 °E)

**Figure 23.**
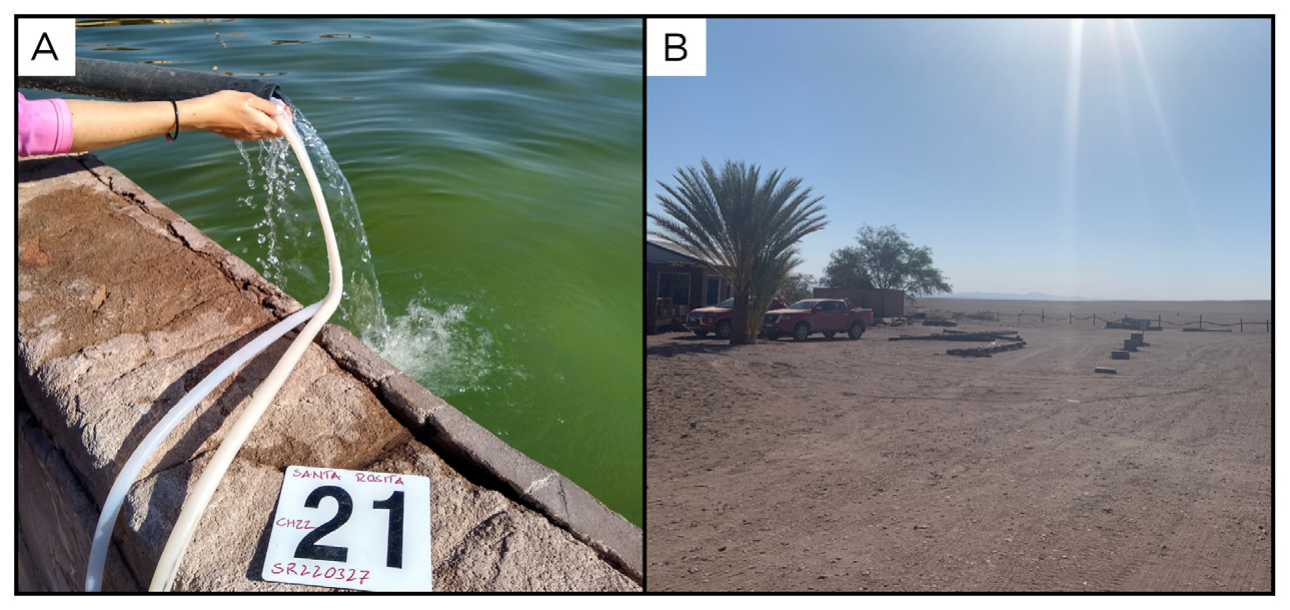
Santa Rosita, SR220328. Fluids were sampled from the water outlet point, near the pool. **A**. Detail of the sampling location where the fluids were collected. **B**. Large view of the sampling site. Photograph taken by author DG for this publication.

Site SR220328 is near the city of Pica, 1322 metres above sea level ( Figure 23). Here the fluids flow from a pipe, supplying a private swimming pool, at 35 ° C. As suggested by the owner of the property, the water is sourced from a nearby natural well. At this site, there is no gas phase, and we did not collect any sediment samples.

### Site 22 - Termas de Chusmiza, CZ220329 (-19.6835 °N, -69.1772 °E)

**Figure 24.**
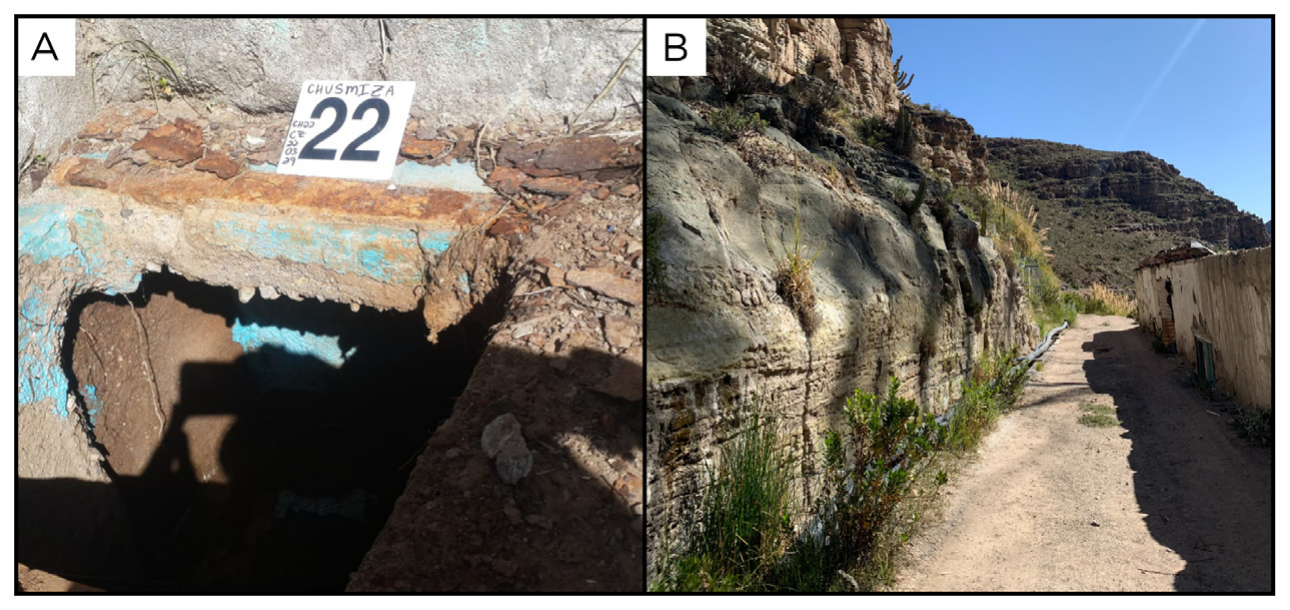
Termas de Chusmiza, CZ220329. Fluids were sampled from the water outlet point. **A**. Detail of the sampling location where the fluids were collected. **B**. Large view of the sampling site. Photograph taken by author DB for this publication.

Site CZ220329 has an altitude of 3423 metres above sea level ( Figure 24). This location has pools with therapeutic thermal water, which serve as an attraction for both residents and tourists. The sampled site is located along a road accessible by car. Fluids were sampled from a concrete tank inside a rock wall, where fluids are 42 ° C. Surrounding the site, thermal water emerges directly from the rock wall.

### Site 23 - Puchuldiza outflow, PZ220330 (-19.4085 °N, -68.9585 °E)

**Figure 25.**
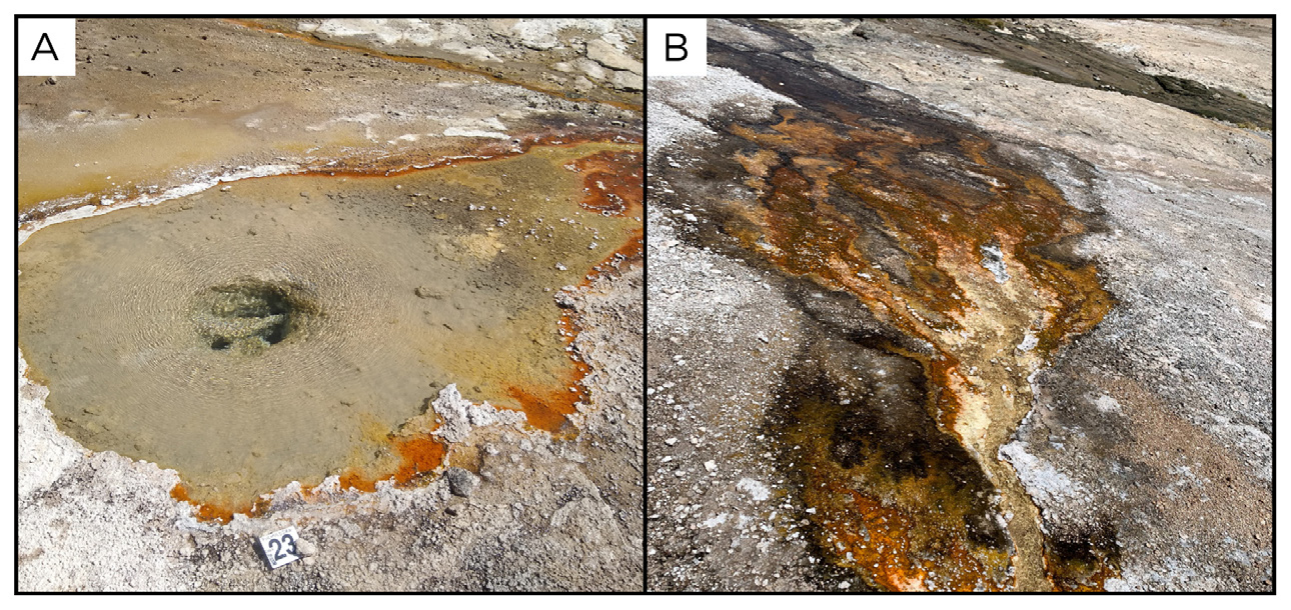
Puchuldiza outflow, PZ220330. Fluids were sampled from the water outlet point. **A**. Detail of the sampling location where the fluids were collected. **B**. Large view of the sampling site. Photograph taken by author DG for this publication.

Site PZ220330 site, 4205 metres above sea level, is approximately 200 metres from the Puchuldiza geothermal field, in the Tarapacá Region ( Figure 25). The spring is located on a dome, at the outer edge of the fumarole/geyser field. Here, fluids have moderate bubbling and a temperature of 74 ° C, flowing downhill in a small stream, with abundant biofilms ranging between green, orange and white, releasing hot fluids into the Puchuldiza River.

### Site 24 - Puchuldiza fumarole, PD220330 (-19.4128 °N, -68.9579 °E)

**Figure 26.**
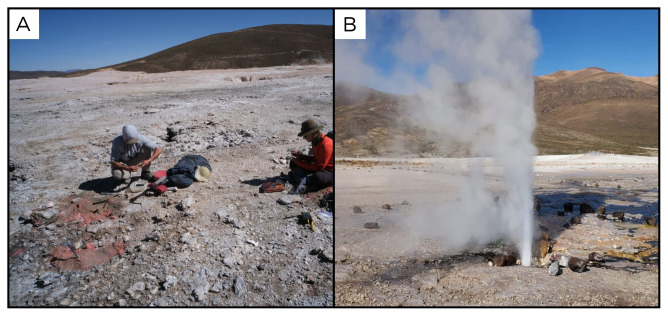
Puchuldiza fumarole, PD220330. **A**. Detail of the sampling location where the gases were collected. **B**. View of a geyser close to the sampling site. Photograph taken by author JP for this publication.

Site PD220330 is in the Puchuldiza geothermal field, in the Tarapacá Region, at an altitude of 4222 metres above sea level ( Figure 26). This area lies within a tectonic graben, and features over a hundred separate geothermal manifestations, such as geysers, fumaroles, and boiling pools. At this site, we measured the temperature at 86 ° C, and we collected condensate samples.

### Site 25 - Parajaya, PJ220330 (-19.1208 °N, -68.9098 °E)

**Figure 27.**
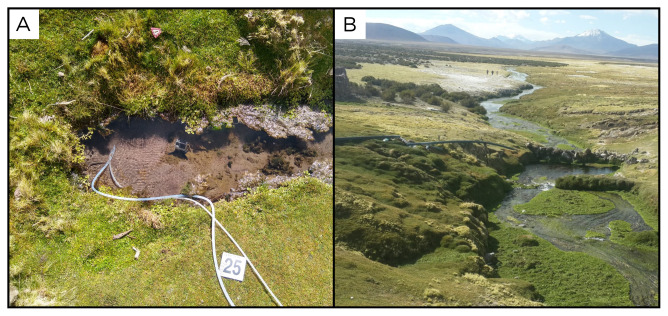
Parajaya, PJ220330. Fluids were sampled from the water outlet point. **A**. Detail of the sampling location where the fluids were collected. **B**. Large view of the sampling site. Photograph taken by author DB for this publication.

Site PJ220330 is close to Río Todos Santos, a river that flows across the Tarapacá Region ( Figure 27). This spring is located in a valley with low vegetation, at an altitude of 4242 metres above sea level, with high water outflow, vegetation, and a temperature of 30 ° C.

### Site 26 - Termas de Enquelga, EQ220330 (-19.2351 °N, -68.7920 °E)

**Figure 28.**
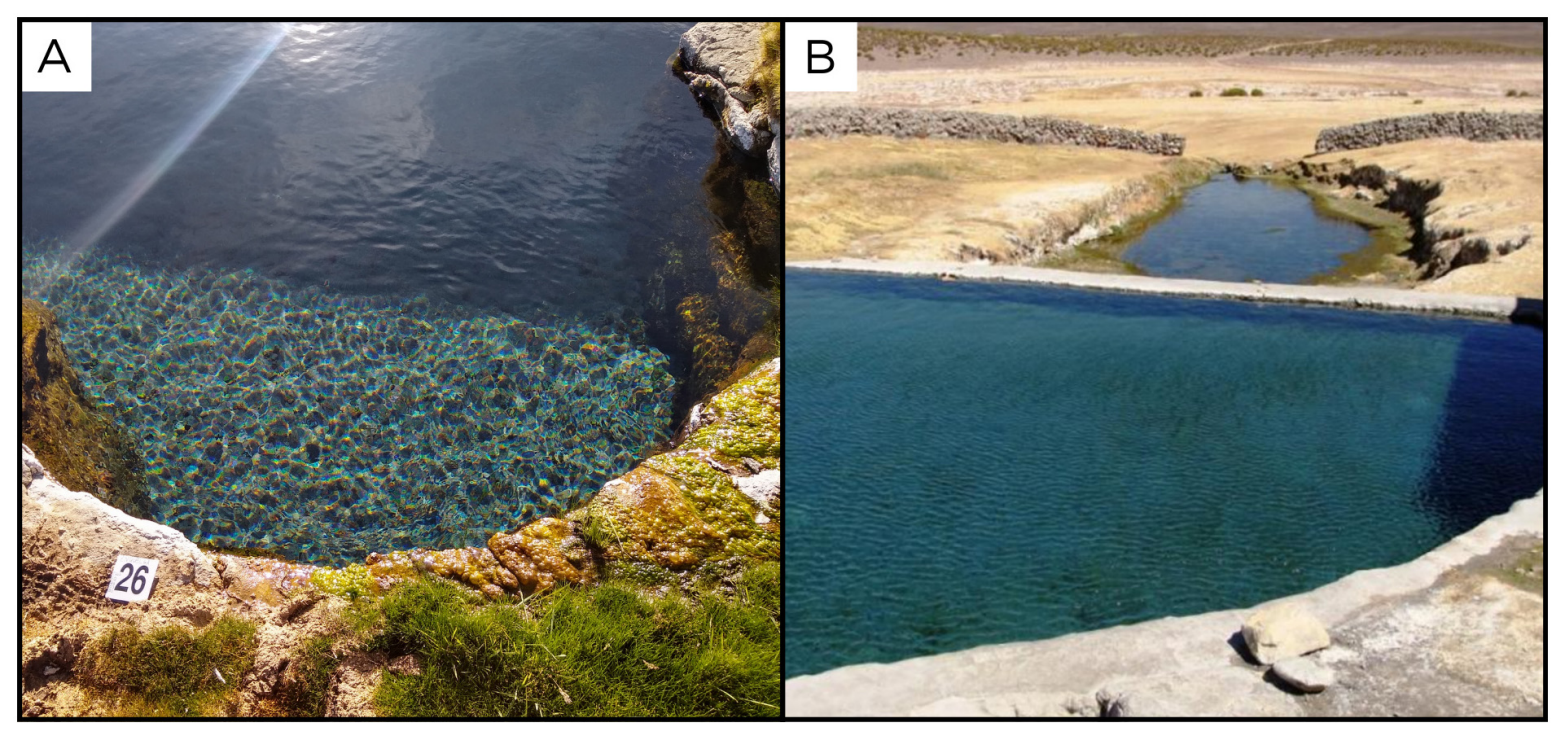
Termas de Enquelga, EQ220330. Fluids were sampled from the water outlet point. **A**. Detail of the sampling location where the fluids were collected. **B**. Large view of the sampling site. Photograph taken by author DB for this publication.

Site EQ220330 is near the small city of Enquelga, at 3901 metres above sea level ( Figure 28). Here, the fluid outlet point is in the wall of a large swimming pool, with high outflow at a temperature of 30 ° C and no gas phase. The pool is frequently used by tourists and locals. After the first swimming pool, the water flows into a second, natural pool.

### Site 27 - Isluga volcano, IV220331 (-19.1622 °N, -68.8349 °E)

**Figure 29.**
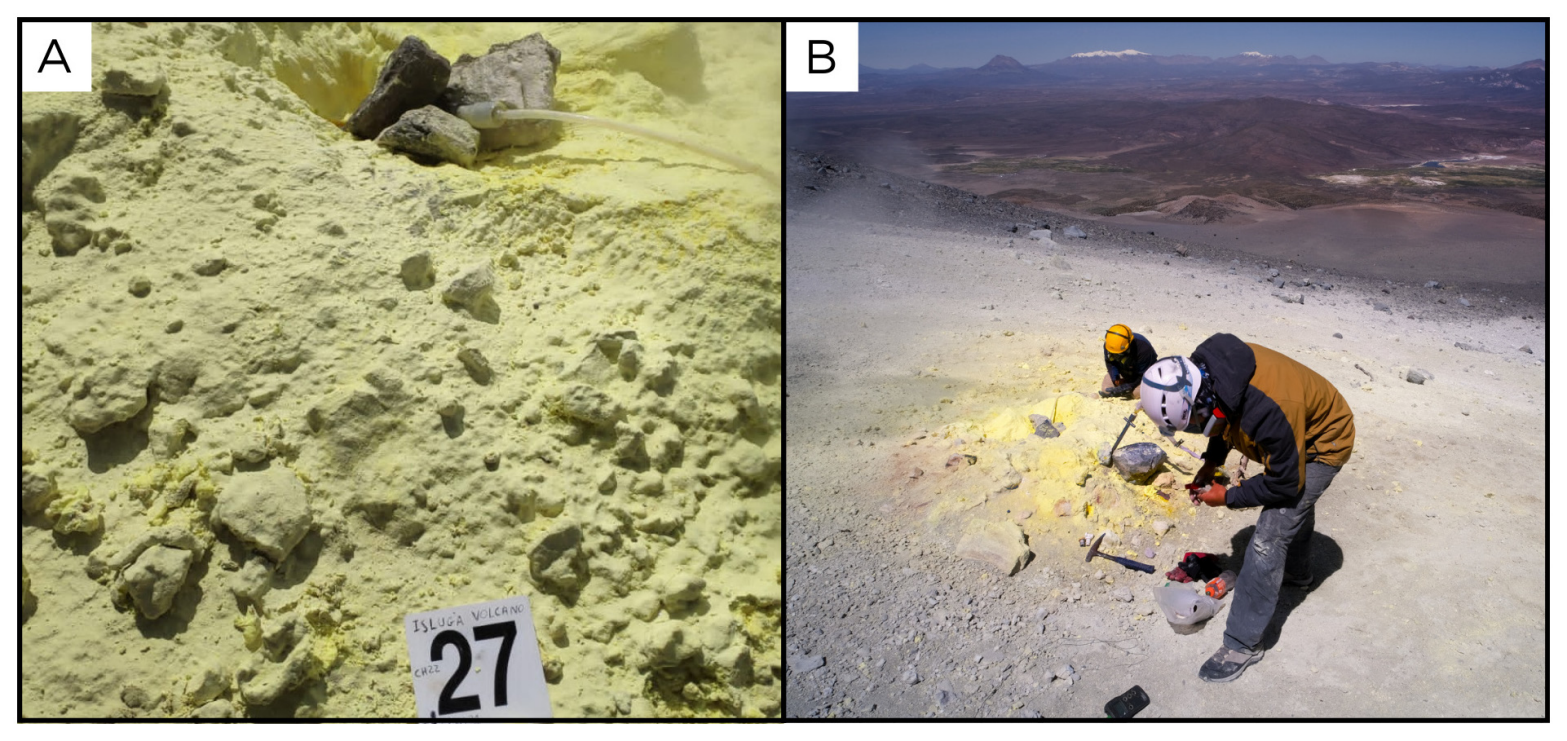
Isluga volcano, IV220331 Fluids were sampled from the water outlet point. **A**. Detail of the sampling location where the fluids were collected. **B**. Large view of the sampling site. Photograph taken by author JP for this publication.

Site IV220331 is a high flux fumarole on the upper flanks of the Isluga stratovolcano, in Colchane, 7 km from the Chilean-Bolivian border, at 5163 metres above sea level ( Figure 29). No fluids were available to be sampled at this site, but we collected condensate samples. The registered temperature at the site where sediments were collected was 97 ° C.

### Site 28 - Terma Tana, TT220331 (-19.1134 °N, -69.1384 °E)

**Figure 30.**
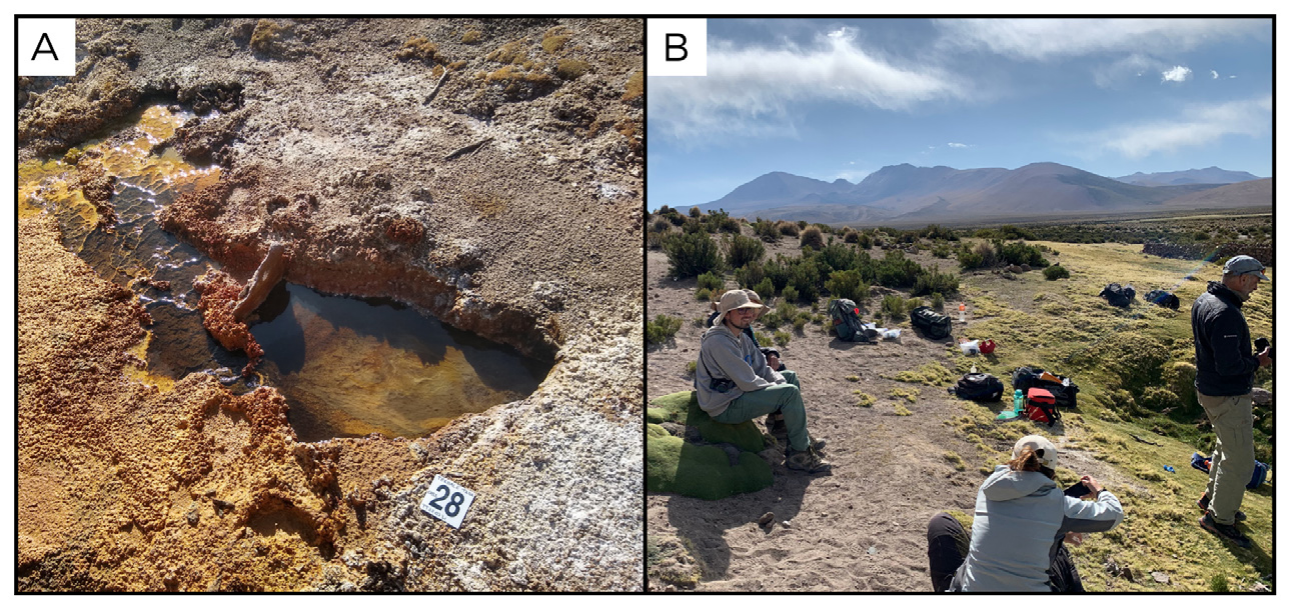
Termal Tana, TT220331. Fluids were sampled from the water outlet point. **A**. Detail of the sampling location where the fluids were collected. **B**. Large view of the sampling site. Photograph taken by author DG for this publication.

Site TT220331 is a hot spring with no gas phase, at 4067 metres above sea level, and a temperature of 67 ° C ( Figure 30). The spring is located on the slope of a small hill, with low vegetation, and is surrounded by carbonate and iron deposits.

### Site 29 - Laguna Amarilla, LA220331 (-19.0588 °N, -69.2528 °E)

**Figure 31.**
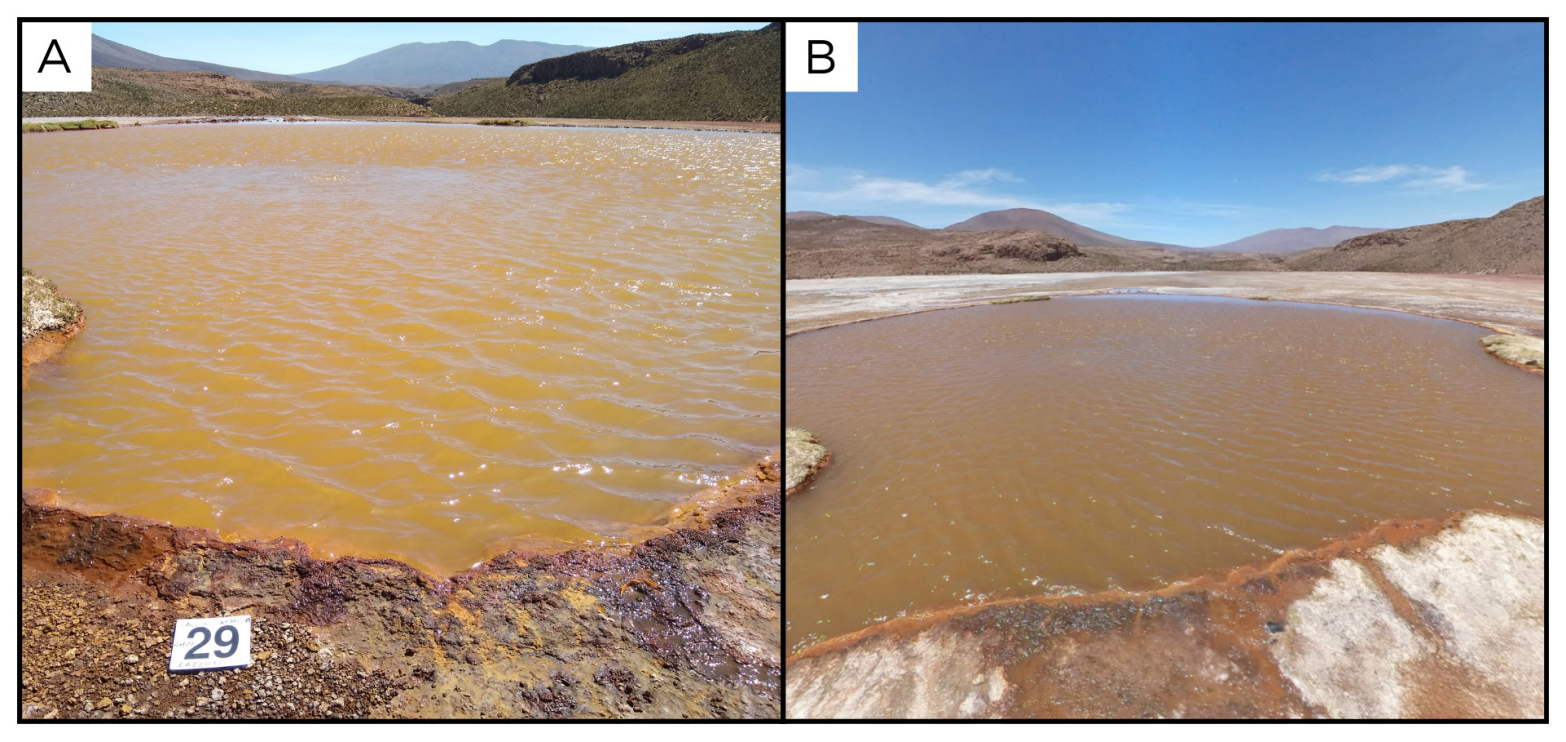
Laguna Amarilla, LA220331. Fluids were sampled from the water outlet point. **A**. Detail of the sampling location where the fluids were collected. **B**. Large view of the sampling site. Photograph taken by author DB for this publication.

Site LA220331 is at the bottom of a 24 m wide lagoon, called Laguna Amarilla, which, together with the Lagura Roja and Laguna Verde, constitute the Lagunas the Amuyo ( Figure 31). These lagunas are located within the Arica y Parinacota Region, and feed the Caritaya river, which flows north, at the base of the dome on which the three lagoons are located. This laguna has an altitude of 3728 metres above sea level, with fluids at 36 ° C. Gas emanates from the centre of the deep pool.

### Site 30 - Laguna Roja, LR220331 (-19.8518 °N, -68.9061 °E)

**Figure 32.**
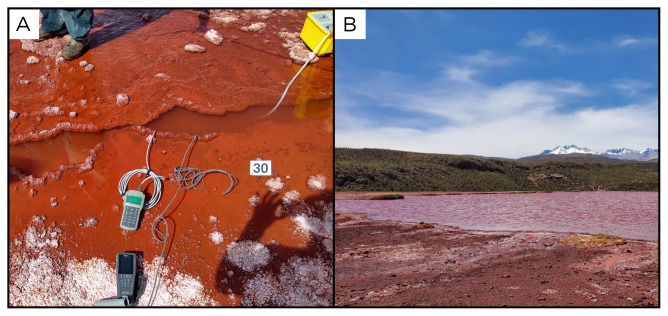
Laguna Roja, LR220331 Fluids were sampled from the water outlet point. **A**. Detail of the sampling location where the fluids were collected. **B**. Large view of the sampling site. Photograph taken by author DG for this publication.

Site LR220331 is on the shores of the Laguna Roja, the second of the three Lagunas the Amuyo, at an altitude of 4079 metres above sea level ( Figure 32). This location stands approximately at the centre of the dome where the three lagunas are located, halfway between the other two. This laguna is a red iron-rich pool with encrustations around edges, with a number of small emissions all around the edges of the pool. We chose one with relatively high water flux, and with a temperature of 24 ° C.

### Site 31 - Laguna Verde, LV220331 (-19.0570 °N, -69.2534 °E)

**Figure 33.**
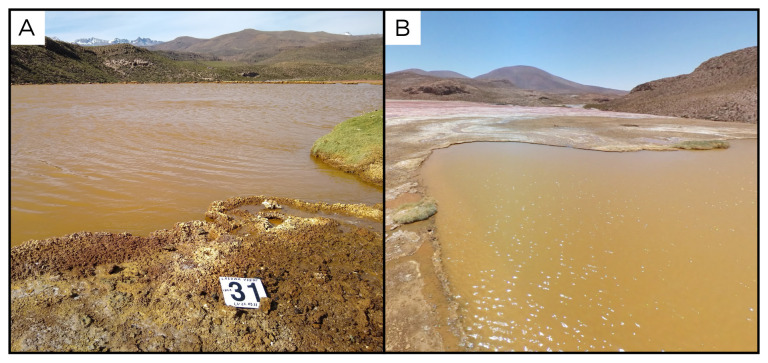
Laguna Verde, LV220331. Fluids were sampled from the water outlet point. **A**. Detail of the sampling location where the fluids were collected. **B**. Large view of the sampling site. Photograph taken by author DG for this publication.

Site LV220331 is on the shore of Laguna Verde, the farthest south of the three Lagunas de Amuyo, at an altitude of 3731 metres above sea level ( Figure 33). The pool is surrounded by encrustations and its outflow ends up in the Caritaya river. Among the three lagunas, this is the warmest, with a temperature of 49 ° C.

### Site 32 - Laguna Parinacota, LP220401 (-19.2330 °N, -69.0104 °E)

**Figure 34.**
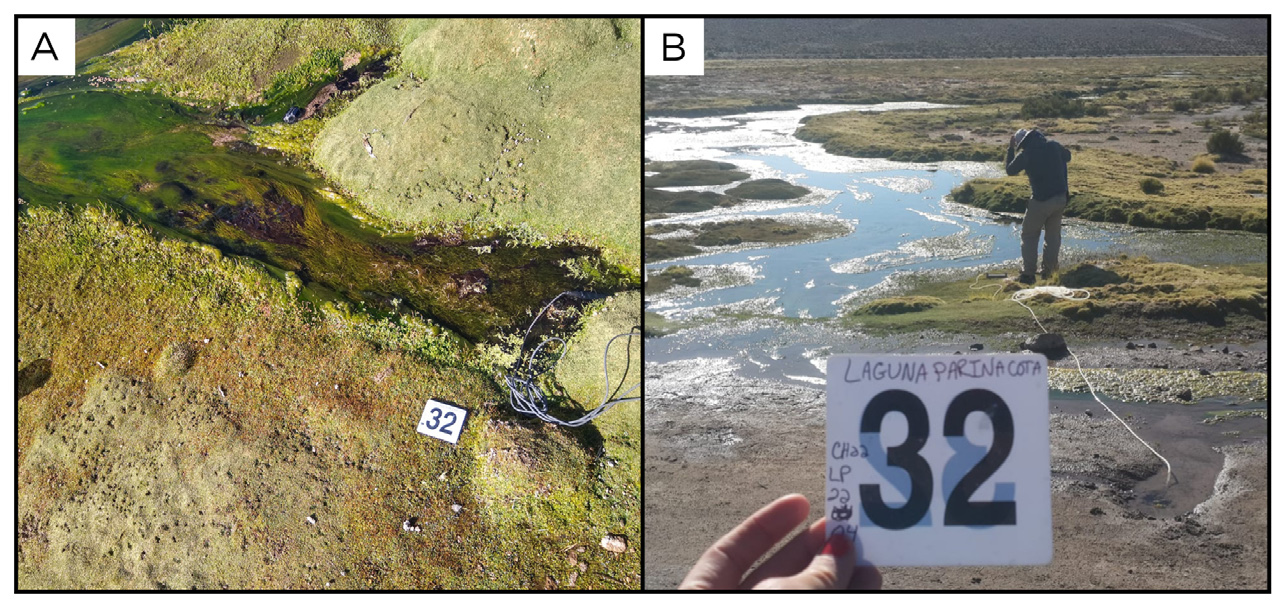
Laguna Parinacota, LP220401. Fluids were sampled from the water outlet point. **A**. Detail of the sampling location where the fluids were collected. **B**. Large view of the sampling site. Photograph taken by author DB for this publication.

Site LP220401, at an altitude of 4157 metres above sea level, is one of two high outflow springs feeding the Laguna Parinacota ( Figure 34). This lagoon is close to the Colchane village and seasonally alternates between dry and flood periods. The site is northwest of the lagoon, on a hillside, with 25 ° C fluids flowing into the lagoon, creating small streams with biofilm and algae.

### Site 33 - Baños Polloquere, PQ220401 (-18.9132 °N, -68.9992 °E)

**Figure 35.**
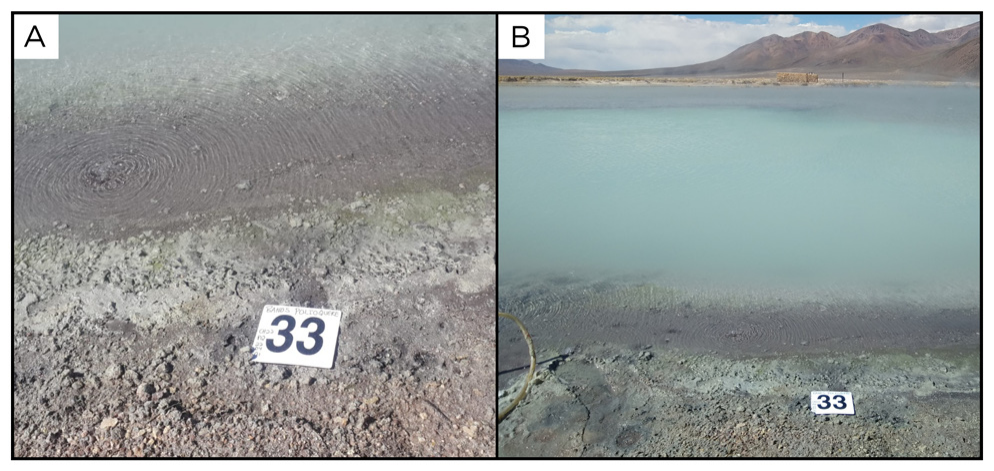
Baño Polloquere, PQ22040. 1 Fluids were sampled from the water outlet point. **A**. Detail of the sampling location where the fluids were collected. **B**. Large view of the sampling site. Photograph taken by author DB for this publication.

Site PQ220401 is located on the shore of a large steaming salt lake, on the southern edge of Salar de Surire, in the Arica y Parinacota Region ( Figure 35). This site has an altitude of 4283 metres above sea level, and is located on one side of the lake, shows strong consistent bubbling and a fluid temperature of 63 ° C.

### Site 34 - Colpitas, CP220402 (-17.9501 °N, -69.4362 °E)

**Figure 36.**
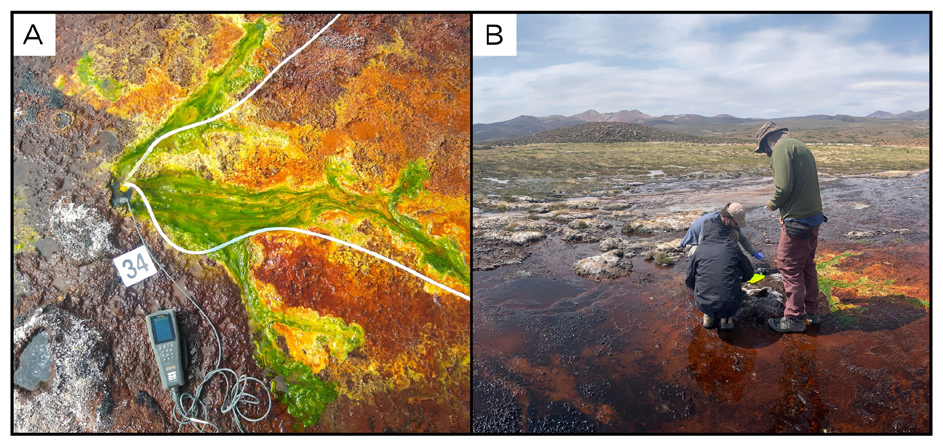
Colpitas, CP220402. Fluids were sampled from the water outlet point. **A**. Detail of the sampling location where the fluids were collected. **B**. Large view of the sampling site. Photograph taken by author JP for this publication.

Site CP220402, at an altitude of 4151 metres above sea level in the Arica y Parinacota Region, is located at the edge of an old inactive geothermal area, with multiple fluid sources, small water streams, and small pools ( Figure 36). With a temperature of 16 °C, this is the spring with the lowest temperature sampled on this expedition and shows very low and intermittent gas bubbles. Fluids flow out of the spring, leaving all around the seep thick microbial mats of different colours, ranging from yellow, orange, brown, and green.

### Site 35 - Colpitas Este, CE220402 (-17.9555 °N, -69.4236 °E)

**Figure 37.**
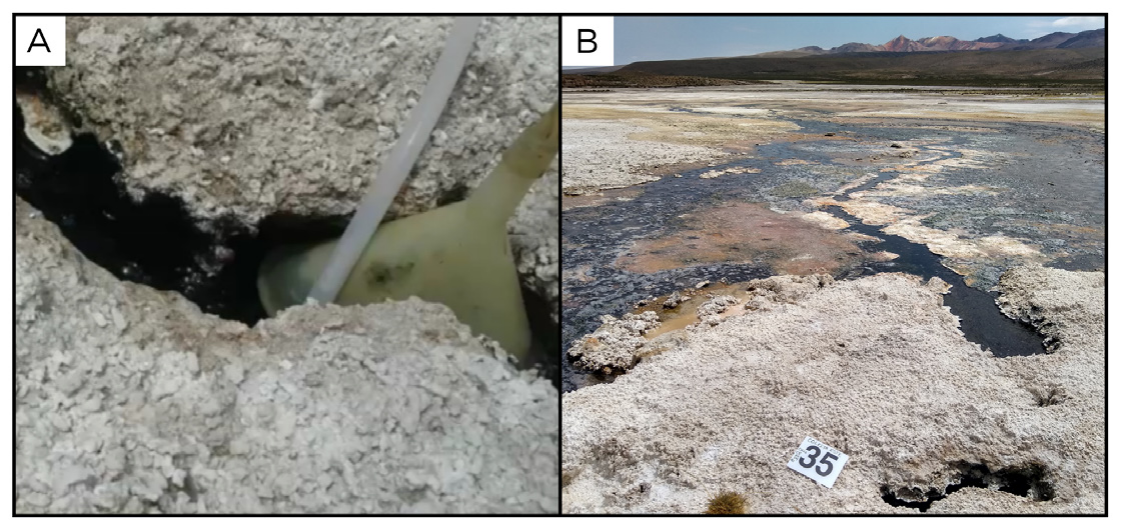
Colpita Este, CE220402. Fluids were sampled from the water outlet point. **A**. Detail of the sampling location where the fluids were collected. **B**. Large view of the sampling site. Photograph taken by author DB for this publication.

Site CE220402 is a warm bubbling spring on the edge of an old inactive geothermal area/salar, located at an altitude of 4141 metres above sea level, in the Arica y Parinacota Region, with 54 °C fluids ( Figure 37). Around the chosen site there are other seeps, which flow into small streams of water across the salar.

### Site 36 - Las Cuevas, CU220402 (-18.1700 °N, -69.4309 °E)

**Figure 38.**
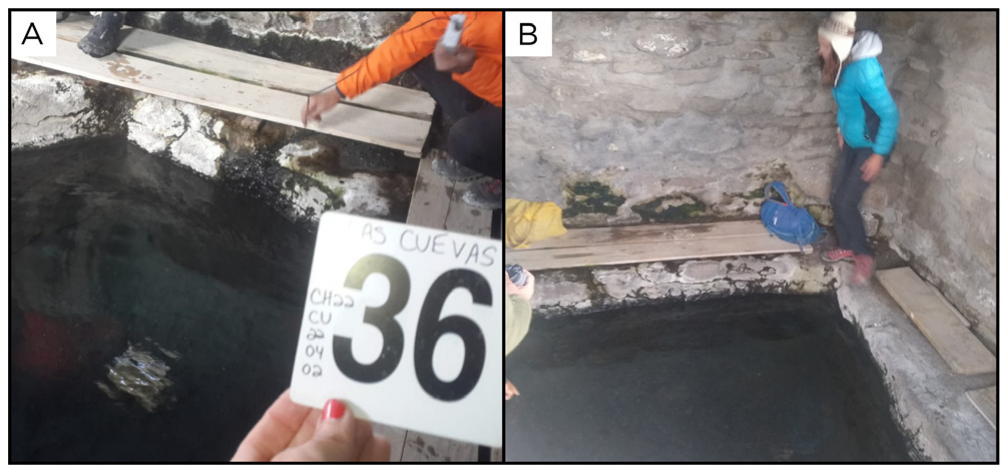
Las Cuevas, CU220402. Fluids were sampled from the water outlet point. **A**. Detail of the sampling location where the fluids were collected. **B**. Large view of the sampling site. Photograph taken by author KGL for this publication.

Site CU220402, also known as Termas de Las Cuevas, is near the location of Las Cuevas, at an altitude of 4473 metres above sea level, with 34 ° C fluids ( Figure 38). The outflow feeds an artificial pool with weak intermittent bubbling within a hut. The water from the spring flows outside the hut, creating a wet area with small streams and abundant biofilms and mats of different colours.

### Site 37 - Putre, PR220403 (-18.1979 °N, -69.5385 °E)

**Figure 39.**
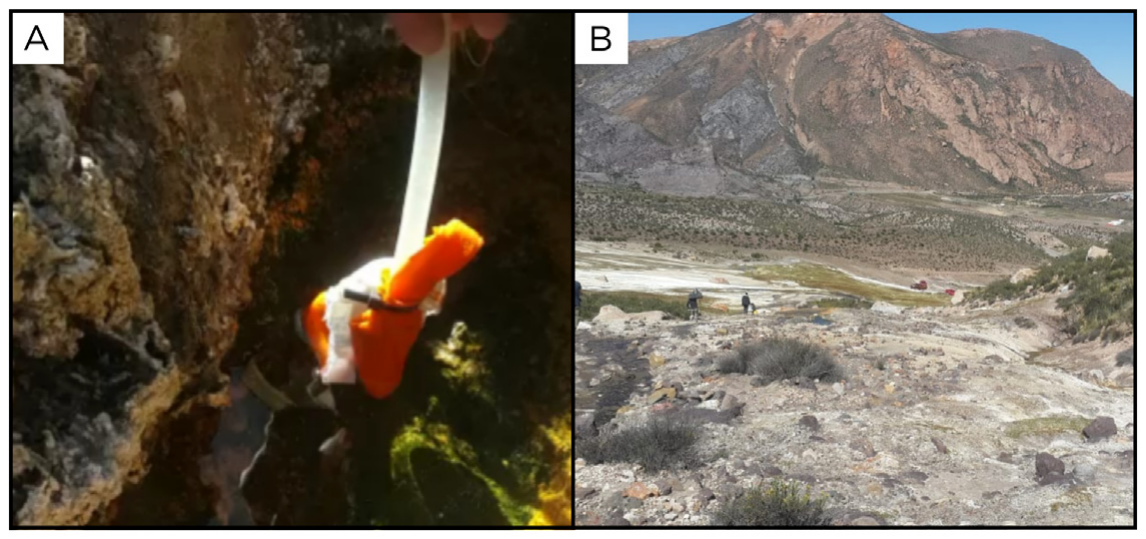
Putre, PR220403. Fluids were sampled from the water outlet point. **A**. Detail of the sampling location where the fluids were collected. **B**. Large view of the sampling site. Photograph taken by author DB for this publication.

Site PR220403 is a small moderately bubbling spring located on the highest point of a large travertine mound, next to the main road ( Figure 39). It has an altitude of 3785 metres above sea level, and fluids flow along the slope of the mound forming a network of small streams at a temperature of 36 ° C. This site was reported by Inostroza *et al.*, 2020 as a Na-Cl type water site.

### Site 38 - Termas de Jurasi, JR220403 (-18.2102 °N, -69.5105 °E)

**Figure 40.**
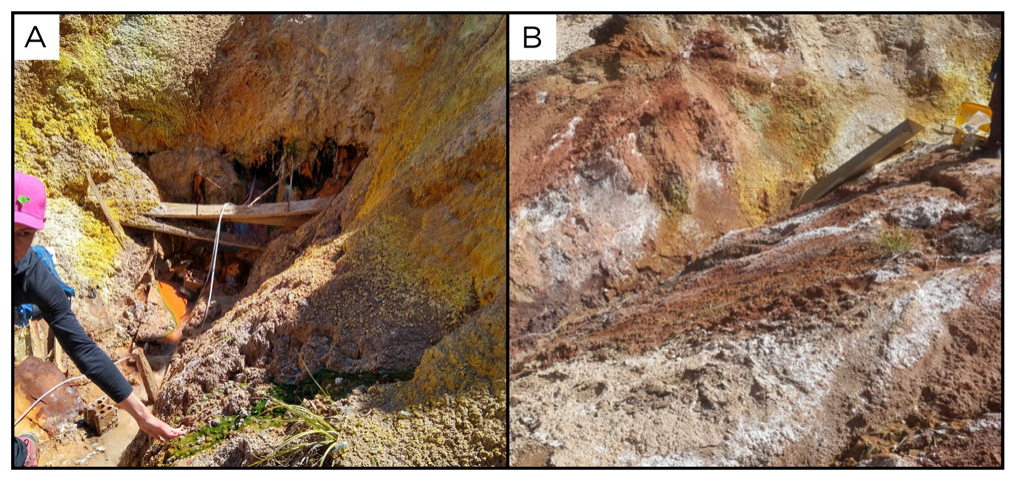
Termas de Jurasi, JR220403. Fluids were sampled from the water outlet point. **A**. Detail of the sampling location where the fluids were collected. **B**. Large view of the sampling site. Photograph taken by author DG for this publication.

JR220403 was the last site sampled in our expedition. This site, at an altitude of 4050 metres above sea level, is a high outflow hot spring located within a private hot springs complex with baths used by tourists and locals ( Figure 40). Here, multiple water sources feed into the pools. Our spring, with a temperature of 65 ° C, doesn’t feed any pool, and flows out from an extremely hydrothermally-altered wall, a few metres above the pools.

## Thermal images of the sampled seeps

**Figure 41.**
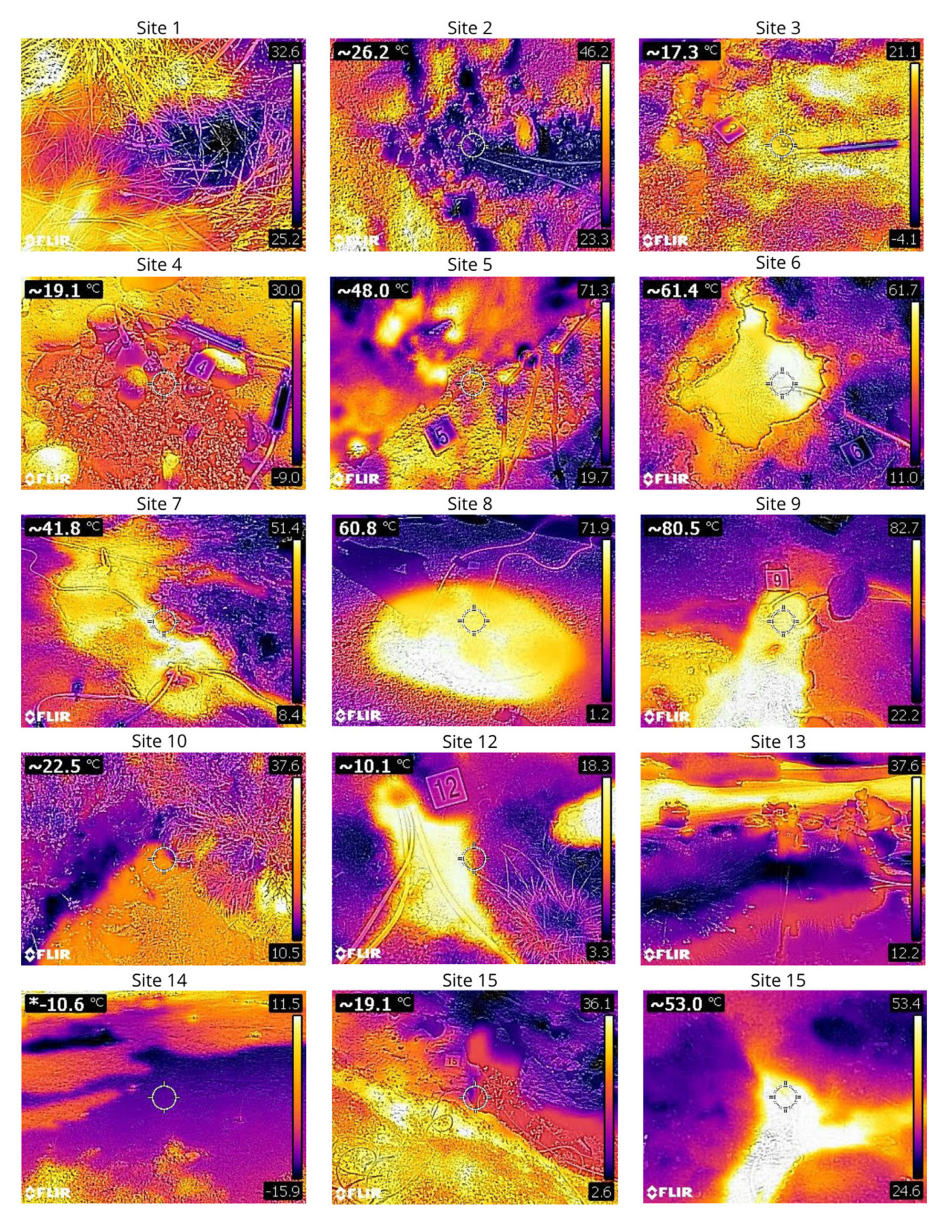
Thermal photos of the sampling sites 1 to 15 taken with a FLIR C2 thermal camera. Photograph taken by author CJR for this publication.

**Figure 42.**
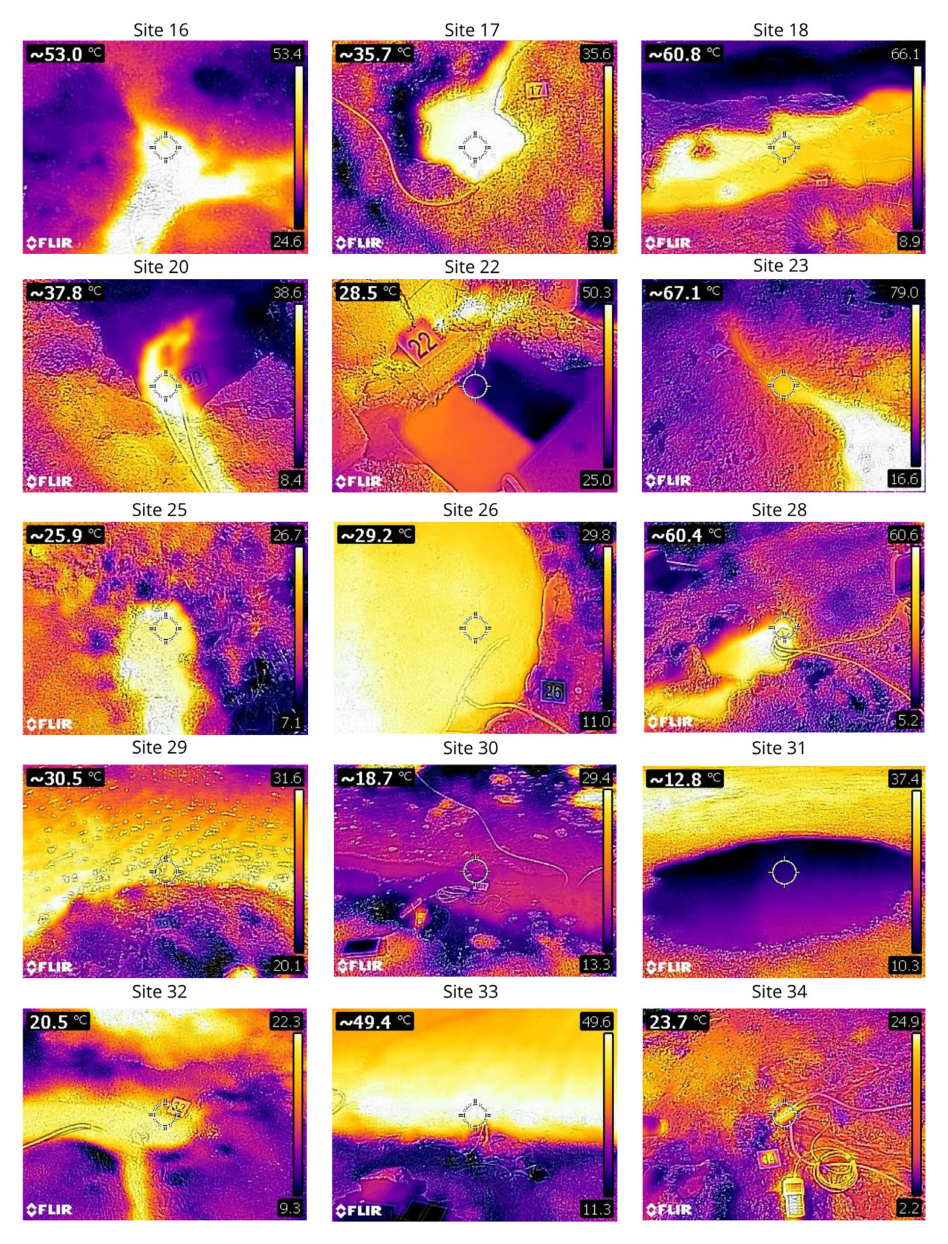
Thermal photos of the sampling sites 16 to 34 taken with a FLIR C2 thermal camera. Photograph taken by author CJR for this publication.

Themal images of the sampled seeps have been collected at each location to assist in sampling site selection and inform on the structure of the seep ( Figure 41 and Figure 42).

## Summary

The expedition successfully sampled 38 sites, including deeply sourced springs and fumaroles. The data obtained will be made public according to the FAIR Principles for scientific data management.

## Ethical approval and consent

Ethical approval and consent were not required.

## Data Availability

The expedition site and metadata are available through a GitHub repository ( https://github.com/giovannellilab/Chile22_Expedition_Report.git) and through Zenodo https://doi.org/10.5281/zenodo.11200954. Zenodo: ERC CoEvolve CHL22 Expedition report. https://doi.org/10.5281/zenodo.11200955 This project contains the following underlying data: CHL22_env_dataset_table1 (1).csv CHL22_Expedition.kmz README.md Data is available under the terms of the Creative Commons Attribution 4.0 International ( https://creativecommons.org/licenses/by/4.0/)
