# Supplementary material for: Targeting deeply-sourced seeps along the Central Volcanic Zone
Source: Open Res Eur. 2024 Oct 18;4:226. [Version 1] doi: 10.12688/openreseurope.17806.1 (PMC11803389; doi:10.12688/openreseurope.17806.1)
Supplement: Peer review history

# Reviewer report: Reviewer response for version 1

**DOI:** 10.21956/openreseurope.19249.r47738

**Published:** 2024-12-19

**Authors:** Francesco Ricci

**Affiliations:** 1 Monash University, Clayton, Victoria, Australia

Bastoni, Giovanelli and colleagues undertook a remarkable sampling effort characterising gas composition, biogeochemistry and microbiology of 38 deeply sourced seeps and fumaroles within the Chilean central volcanic zone. The manuscript is logically organised and well written. The amount of data generated through this expedition will deliver an important milestone to the scientific literature and expand our understanding of the process at the intersection between geology and biology.

**Detailed comments**

Abstract

- The knowledge gap that this project is trying to fill is still hidden, I suggest highlighting it better.
- Please expand on the implications that this study will have for our understanding of the processes that have shaped Earth’s biogeochemistry and biology.

Introduction

- At the end of the introduction, I would reiterate the rationale for performing this study, the knowledge gaps it will fill and the methodologies that will be used to investigate the data.  

Methods – Biological samples acquisition

- Please clarify how the peristaltic pump was sterilised between sampling sites and/or how cross-contamination precautions were taken.
- Perhaps expand on what sort of omics the microbiology samples will be used for.
- Line 6 – change from ‘through an battery’ to ‘through a battery’.

Are sufficient details of methods and materials provided to allow replication by others?

Yes

Is the rationale for creating the dataset(s) clearly described?

Yes

Are the datasets clearly presented in a useable and accessible format?

Yes

Are the protocols appropriate and is the work technically sound?

Yes

Reviewer Expertise:

Microbiology, microbial ecology, biogeochemistry

I confirm that I have read this submission and believe that I have an appropriate level of expertise to confirm that it is of an acceptable scientific standard.

# Reviewer report: Reviewer response for version 1

**DOI:** 10.21956/openreseurope.19249.r47123

**Published:** 2024-12-19

**Authors:** Magdalena Osburn, Dylan Chambers

**Affiliations:** 1 Northwestern University, Evanston, Illinois, USA

Summary: This data report describes a large-scale sampling campaign and resultant sample set targeting springs and fumaroles in the central volcanic zone of Chile. They document the field context, samples collected, and basic metadata of each site and include site thermal infrared images. This collection would not warrant a full publication elsewhere, but in the spirit of open data and allowing the authors to cite the field descriptions in future work, I see the value of this contribution. I would, however, like to see a small amount of additional information and some slight reorganization.

 Common points:

 For a data report, there is a pretty limited amount of data included here which could be used elsewhere. Others may use this document to replicate sampling locations, but to use the report in that way it needs more information.

1. To replicate this sampling process, it is imperative to know more about where in each spring was sampled. While parameters are described in the methods about what would be optimal, it is important to know where was actually sampled for each site. Specifically: how close to the source was water sampled?, how deep was the water there?, where and how far from the source was each background contextual sample taken?
2. In the site context photos it is only possible know where exactly the sample was taken in those with tubing visible. It is often challenging to relate the close up picture to the overview as they are from very different angles. I suggest adding arrows to both images to indicate the sampling location.

Since this publication is aiming to make this science more open, I think documentation of what (if any) sample material might be available for other researchers who might not be able to mount an expedition like this. The methods report what was taken, they could also include what was consumed in sample processing for subsequent publications, and thus indicate what is left and where it resides.

 Formatting suggestions:

 -There are inconsistencies in how many spaces surround degree signs. These should always be next to the C, but may or may not have a space between the number and the unit depending on followed convention. Regardless, it should be consistent throughout.

 -please define an abbreviation for “meters above sea level”, writing this out 34X gets redundant.

 Minor points:

 Abstract:

 -the opening line is unnecessarily broad and not strictly correct

 Expedition team and logistics:

 -“deeply sourced seeps” – this seems like something that would need to be demonstrated. How do we know that these are deeply sourced?

 Geological… settings

 -“The last Andean…” I am confused about the chronology here. If it is relevant to the dataset described here, please walk through the chronology more clearly

 Methods:

 -Sentence on permissions could just say “ we obtained permission from land owners prior to sampling”

 - “nearby area” – this is vague

 Page 6

 -Presumably the falcons were rinsed after acid cleaning?

 -The sentence on background soil sampling. Can you give a range of how far way those were from each site? How was it determined that those sites weren’t subjected to hydrothermal activity? As written, this could be very subjective. I would suggest adding this distance to each site paragraph where relevant.

 Page 11

 Site 10 – is 25’C a hot spring by definition? I don’t think so.

 Figures:

 All figures:

 -There are not letters annotated on the images so the reader must assume which is A, B, C, etc.

 -It would be helpful to have the site # in the figure captions.

 -The figure captions are very generic, but different views and content appear on the photographs. It would be helpful if these were more tailored to describe specifically what is being shown at each site.

 -I would suggest grouping images to put those from similar areas together. This might help the reader understand better which areas are related vs. distant. I recognize that some will be alone, but for instance “the lagunas” could be a 6 panel figure easily.

 Figure 5A – is this a drone image? It would be good to mention specify.

 -Figure 1 – I don’t see a rectangle on A and for C, these are not geological units but rather rock types.

 -Figure 2 – It would be more accurate to describe the alkalinity as total alkalinity (which you measured directly) rather than as CaCO3, which requires assumptions about other ions.

 -Figure 22 – There is only one photo here. No A

Are sufficient details of methods and materials provided to allow replication by others?

No

Is the rationale for creating the dataset(s) clearly described?

Partly

Are the datasets clearly presented in a useable and accessible format?

Yes

Are the protocols appropriate and is the work technically sound?

Yes

Reviewer Expertise:

Geomicrobiology, extreme environments, isotope geochemistry

We confirm that we have read this submission and believe that we have an appropriate level of expertise to confirm that it is of an acceptable scientific standard, however we have significant reservations, as outlined above.

# Reviewer report: Reviewer response for version 1

**DOI:** 10.21956/openreseurope.19249.r47121

**Published:** 2024-12-13

**Authors:** Mark Alexander Lever

**Affiliations:** 1 University of Texas at Austin, Port Aransas, USA

This study describes the geomicrobiological sampling effort that went into the sampling of 38 deeply-sourced seeps along the Central Volcanic Zone. The bulk of the funding came from an ERC grant to the first author Dr. Donato Giovannelli. The sampling campaign was carried out by a large international team of mostly European, South and North American collaborators.

 The manuscript is well-organized and well-written, and provides fantastic photographic documentation of the sampling sites. The reader is left hungry for the scientific results of this unprecedented sampling effort, which with careful integrations of microbiological, geochemical, and geophysical analyses is almost guaranteed to produce a landmark study on geosphere-biosphere interactions in tectonically active regions.

 I only have a few minor comments, mostly concerning the Abstract and Introduction.

 Abstract:

 Please shorten the introductory text and instead please expand the text on your project. E.g, what kinds of protocols did you develop? Broadly, what were the (ranges of) environmental characteristics of the sites that were sampled? What is the broader importance of the overall project, and of this manuscript (e.g., so the reader understands why you did not publish the info contained in this manuscript in a study that contains scientific results).

 Also, a minor comment regarding the slightly misleading first sentence: subduction only occurs at convergent margins where an oceanic plate collides with a terrestrial plate (but not at convergent margins of bordering terrestrial plates).

 Introduction

 The first paragraph covers a lot of information, without explaining it. For readers who are not familiar with the field, please briefly explain how the "intricate interplay between the descending slab, the overlying mantle wedge, and the overriding lithosphere, collectively exert a profound influ- ence on the thermal and volatile fluxes produced, the nature of the underlying rock formations, and their interactions with percolating fluids, among other factors"

 minor comments:

 L. 5: not clear what is meant with "manifestations"

 L. 15/16: not clear what is meant with "these changes"

 Figure 1: 

 Please show the distributions of the NVZ, CVZ, SVZ, and AVZ in the map of South America

 Methods:

 1st sentence: "We collected gases, fluids, and sediments from fumaroles and deeply-sourced seeps" --> you need to devote an entire paragraph to this. Please include:

 - why did you choose these sites (and seeps)? What makes them suitable/interesting for the project (e.g. what poly-extremes do they include?)

 - what samples were taken for which analyses?

 - what question(s) do these samples and analyses help you address? You later on mention the analyses you did, but not why you did them (e.g. what questions they help address)

 - how do you know that these seeps are "deeply sourced"?

 In the Abstract you write "We carried out a scientific expedition, sampling along a ~800 km convergent margin segment of the Andean Convergent Margin in the Central Volcanic Zone of northern Chile, between 17 °S and 24 °S, sampling fluids, gases and sediments, in an effort to understand interactions between microbiology, deeply-sourced fluids, the crust, and tectonic parameters."

 Based on the text, I am not clear about your strategy to tackle this complex multidisciplinary question. Please explain - in a few sentences - how your project - from the choice of sampling locations to the samples that were taken to the background information that you already had on the "crust, and tectonic parameters" - addresses this question.

 Figure 2

 Why are you showing this figure? You are not showing any other scientific results, so why this one? What does this figure tell us?

Are sufficient details of methods and materials provided to allow replication by others?

Yes

Is the rationale for creating the dataset(s) clearly described?

Yes

Are the datasets clearly presented in a useable and accessible format?

Yes

Are the protocols appropriate and is the work technically sound?

Yes

Reviewer Expertise:

Sediment biogeochemistry, geomicrobiology, microbial ecology, carbon cycle, deep biosphere, life under extreme energy limitation

I confirm that I have read this submission and believe that I have an appropriate level of expertise to confirm that it is of an acceptable scientific standard.
